# Association between fetal abdominal growth trajectories, maternal metabolite signatures early in pregnancy, and childhood growth and adiposity: prospective observational multinational INTERBIO-21st fetal study

**DOI:** 10.1016/S2213-8587(22)00215-7

**Published:** 2022-10

**Authors:** Jose Villar, Roseline Ochieng, Robert B Gunier, Aris T Papageorghiou, Stephen Rauch, Rose McGready, Julia M Gauglitz, Fernando C Barros, Manu Vatish, Michelle Fernandes, Victor Zammit, Verena I Carrara, Shama Munim, Rachel Craik, Hellen C Barsosio, Maria Carvalho, James A Berkley, Leila I Cheikh Ismail, Shane A Norris, Chrystelle O O Tshivuila-Matala, Francois Nosten, Eric O Ohuma, Alan Stein, Ann Lambert, Adele Winsey, Ricardo Uauy, Brenda Eskenazi, Zulfiqar A Bhutta, Stephen H Kennedy

**Affiliations:** a Nuffield Department of Women's & Reproductive Health, University of Oxford, Oxford, UK; b Oxford Maternal & Perinatal Health Institute, Green Templeton College, University of Oxford, Oxford, UK; c Centre for Tropical Medicine and Global Health, Nuffield Department of Medicine, University of Oxford, Oxford, UK; d Wellcome Centre for Human Genetics, Nuffield Department of Medicine, University of Oxford, Oxford, UK; e Department of Psychiatry, University of Oxford, Oxford, UK; f Faculty of Health Sciences, Aga Khan University, Nairobi, Kenya; g Centre of Excellence in Women and Child Health, Aga Khan University, Nairobi, Kenya; h Center for Environmental Research and Community Health, School of Public Health, University of California, Berkeley, CA, USA; i Shoklo Malaria Research Unit, Mahidol-Oxford Tropical Medicine Research Unit, Faculty of Tropical Medicine, Mahidol University, Mae Sot, Thailand; j Sapient Bioanalytics, San Diego, CA, USA; k Programa de Pós-Graduação em Saúde e Comportamento, Universidade Católica de Pelotas, Pelotas, Brazil; l Medical Research Council Lifecourse Epidemiology Centre & Human Development and Health Academic Unit, Faculty of Medicine, University of Southampton, Southampton, UK; m Biomedical Sciences, Translational & Experimental Medicine, Warwick Medical School, University of Warwick, Coventry, UK; n Department of Obstetrics and Gynaecology, Division of Women and Child Health, Aga Khan University, Karachi, Pakistan; o Kenya Medical Research Institute-Coast Centre for Geographical Medicine and Research, University of Oxford, Kilifi, Kenya; p Department of Obstetrics & Gynaecology, Faculty of Health Sciences, Aga Khan University Hospital, Nairobi, Kenya; q Clinical Nutrition and Dietetics Department, University of Sharjah, Sharjah, United Arab Emirates; r South African Medical Research Institute Developmental Pathways For Health Research Unit, Department of Paediatrics & Child Health, University of the Witwatersrand, Johannesburg, South Africa; s Medical Research Council and Wits Rural Public Health and Health Transitions Research Unit (Agincourt), School of Public Health, Faculty of Health Sciences, University of the Witwatersrand, Johannesburg, South Africa; t African Health Research Institute, KwaZulu-Natal, South Africa; u Health, Nutrition & Population Global Practice, World Bank Group, Washington, DC, USA; v Maternal, Adolescent, Reproductive & Child Health Centre, London School of Hygiene & Tropical Medicine, London, UK; w Department of Nutrition and Public Health Interventions Research, London School of Hygiene and Tropical Medicine, London, UK; x Center for Global Child Health, Hospital for Sick Children, Toronto, ON, Canada

## Abstract

**Background:**

Obesity predominantly affects populations in high-income countries and those countries facing epidemiological transition. The risk of childhood obesity is increased among infants who had overweight or obesity at birth, but in low-resource settings one in five infants are born small for gestational age. We aimed to study the relationships between: (1) maternal metabolite signatures; (2) fetal abdominal growth; and (3) postnatal growth, adiposity, and neurodevelopment.

**Methods:**

In the prospective, multinational, observational INTERBIO-21st fetal study, conducted in maternity units in Pelotas (Brazil), Nairobi (Kenya), Karachi (Pakistan), Soweto (South Africa), Mae Sot (Thailand), and Oxford (UK), we enrolled women (≥18 years, with a BMI of less than 35 kg/m^2^, natural conception, and a singleton pregnancy) who initiated antenatal care before 14 weeks’ gestation. Ultrasound scans were performed every 5±1 weeks until delivery to measure fetal growth and feto–placental blood flow, and we used finite mixture models to derive growth trajectories of abdominal circumference. The infants’ health, growth, and development were monitored from birth to age 2 years. Early pregnancy maternal blood and umbilical cord venous blood samples were collected for untargeted metabolomic analysis.

**Findings:**

From Feb 8, 2012, to Nov 30, 2019, we enrolled 3598 pregnant women and followed up their infants to 2 years of age. We identified four ultrasound-derived trajectories of fetal abdominal circumference growth that accelerated or decelerated within a crucial 20–25 week gestational age window: faltering growth, early accelerating growth, late accelerating growth, and median growth tracking. These distinct phenotypes had matching feto–placental blood flow patterns throughout pregnancy, and different growth, adiposity, vision, and neurodevelopment outcomes in early childhood. There were 709 maternal metabolites with positive effect for the faltering growth phenotype and 54 for the early accelerating growth phenotype; 31 maternal metabolites had a negative effect for the faltering growth phenotype and 76 for the early accelerating growth phenotype. Metabolites associated with the faltering growth phenotype had statistically significant odds ratios close to 1·5 (ie, suggesting upregulation of metabolic pathways of impaired fetal growth). The metabolites had a reciprocal relationship with the early accelerating growth phenotype, with statistically significant odds ratios close to 0.6 (ie, suggesting downregulation of fetal growth acceleration). The maternal metabolite signatures included 5-hydroxy-eicosatetraenoic acid, and 11 phosphatidylcholines linked to oxylipin or saturated fatty acid sidechains. The fungicide, chlorothalonil, was highly abundant in the early accelerating growth phenotype group.

**Interpretation:**

Early pregnancy lipid biology associated with fetal abdominal growth trajectories is an indicator of patterns of growth, adiposity, vision, and neurodevelopment up to the age of 2 years. Our findings could contribute to the earlier identification of infants at risk of obesity.

**Funding:**

Bill & Melinda Gates Foundation.

## Introduction

Obesity affects populations in most high-income countries and those undergoing an epidemiological transition,^1^ with women of reproductive age and children overrepresented.^2^ The risk of childhood obesity is increased if an infant already has overweight or obesity at birth.^3^ Conversely, in low-income and middle-income countries, one in five infants are born small for gestational age (SGA).^4^ The rate of weight gain for infants in the first 2 postnatal years establishes early childhood total and abdominal fat mass, and cardiometabolic disease risk in later life.^5^

There is increasing evidence that global SGA rates are associated more with the maternal exposome, including pre-pregnancy and intergenerational effects, than genetic factors alone,^6^ because growth and neurodevelopmental patterns from conception to early childhood are similar across geographically diverse populations that have adequate health, nutrition, education, and breastfeeding.^7^, ^8^ Thus, the complexity of size at birth and its influence on later life are best understood by considering how the maternal exposome influences the biology of early human growth and development.^9^

**Research in context**

**Evidence before this study**

We searched, without any language restriction, Embase, PubMed, Cochrane, LILACS, NIH, and NHS, from Jan 1, 2001, to Aug 31, 2021, for “metabolomics”, “metabolites”, “metabolome”, “proteomics”, “proteome”, and “small for gestational age”, “intrauterine growth restriction”, “impaired fetal growth” and “maternal morbidity”. Google Scholar, proceedings of perinatal congresses, references of identified studies, and previous reviews were also searched. This search revealed one cohort and five case–control studies, all with small sample sizes. Of these, four reported associations with small for gestational age (SGA), including (1) a 19-metabolite signature that included phosphatidylcholines and fatty acids; (2) glycerophospholipids, including phosphatidylcholines; (3) eicosanoids, some of which were negatively associated with large for gestational age; and (4) a ratio of two positively and two negatively associated metabolites that predicted intrauterine growth restriction at term.

**Added value of this study**

To facilitate a more comprehensive evaluation of how the maternal exposome influences the biology of early human growth and development, we analysed the relationships between size at birth and: maternal metabolite signatures (as a measure of the exposome); four fetal phenotypes (ultrasound-derived, abdominal circumference trajectories, and their feto–placental blood flow patterns); and postnatal growth, adiposity, and neurodevelopment. These distinct fetal phenotypes had different growth, adiposity, vision, and neurodevelopment outcomes in early childhood. The extreme phenotypes (faltering growth and early accelerating growth) had a significant reciprocal relationship with a set of early-pregnancy maternal metabolite signatures: 5-hydroxy-eicosatetraenoic acid and 11 phosphatidylcholines linked to oxylipin or saturated fatty acid side-chains.

**Implications of all the available evidence**

Understanding how the maternal exposome influences early human development requires deep phenotyping to complement clinical classifications of complex perinatal syndromes, such as SGA and preterm birth. We have identified putative metabolic pathways responsible for the strong association between distinct trajectories of fetal growth and childhood health outcomes. Routine identification of the described trajectories for abdominal circumference, in addition to head circumference trajectories, and the timing of their divergence, should be incorporated into the diagnosis of fetal growth restriction.

Here, we aimed to study, in a large multinational pregnancy cohort, the relationships between: (1) maternal metabolite signatures (as a measure of the exposome); (2) ultrasound-derived fetal abdominal circumference trajectories and their feto–placental blood flow patterns; and (3) postnatal growth, adiposity, and neurodevelopment.

## Methods

### Study design and participants

The prospective, multinational, observational INTERBIO-21st fetal study was conducted in maternity units in Pelotas (Brazil), Nairobi (Kenya), Karachi (Pakistan), Soweto (South Africa), Mae Sot (Thailand), and Oxford (UK) between Feb 8, 2012, and Nov 30, 2019**.**^10^ We enrolled 3598 women who initiated antenatal care before 14 weeks’ gestation, identified by ultrasound dating,^11^ and monitored their pregnancies to delivery. Their children's health, growth, and development were monitored until the age of 2 years; a corrected age was used for infants who were born preterm. The inclusion criteria for the women were: being 18 years or older, a BMI of less than 35 kg/m^2^, natural conception, and singleton pregnancy. A sample of non-fasting venous blood was obtained at the earliest possible opportunity, at a median (IQR) gestational age of 13·2 weeks (11·9–17·2), plus a sample of umbilical cord venous blood at delivery; all samples were processed and stored as per protocol.^12^

The study was approved by the Oxfordshire Research Ethics Committee, the research ethics committees of the participating institutions, and their regional health authorities. All mothers provided written informed consent.

### Procedures

After the dating ultrasonography, the women underwent an ultrasonography every 5±1 weeks until delivery using identical equipment (Philips HD-9, Philips Ultrasound, Andover, MA, USA). The methods to measure abdominal circumference growth have previously been described.^13^ From 22 weeks’ gestation, the umbilical artery Doppler Pulsatility Index, a marker of feto–placental perfusion, was measured. Mean umbilical artery Doppler Pulsatility Index values were expressed as z-scores of the international standard (reference).^14^ The anthropometric measurement methods used have previously been described.^15^ Infant age-specific and sex-specific z-scores and centiles were compared with the WHO Child Growth Standards.^16^

We assessed neurodevelopment at the age of 2 years using the INTERGROWTH-21st Neurodevelopmental Assessment (INTER-NDA), a multicultural, psychometric tool for children aged 22–30 months, designed to be implemented by non-specialists across international settings, which measures multiple dimensions of early development using directly administered, concurrently observed, and caregiver reported items.^17^ Metabolite signatures were measured using untargeted mass spectrometry (Sapient Bioanalytics, San Diego, CA, USA) and compound identification was performed by spectral matching to open access and private spectral databases. To visualise the metabolite feature data, we used Uniform Manifold Approximation and Projection (UMAP) with the Python package UMAP-learn.^18^ For more details of the metabolomic analysis, see the appendix (pp 3–5).

### Statistical analysis and outcomes

To establish the abdominal circumference growth trajectories (the primary outcome of the study), we constructed models for the repeated measurement of abdominal circumference z-scores using finite mixture models.^19^ Group mean growth patterns were modelled using Gaussian distributions by applying a quadratic B-spline with one internal knot placed at the median.^20^ A z-score of 0 at 10 weeks’ gestation for each infant was added to highlight the growth trajectories relative to the initial ultrasonography and prevent grouping strictly by size. All mixture modelling was done using the hlme function of the R package lcmm.^21^, ^22^

Posterior group probabilities for models with three to five growth pattern groups were estimated,^23^ allowing for a low number of trajectories following recognised fetal growth patterns. Trajectories were established using only fetal measures without considering their 2-year outcomes or metabolomic data, to which the statistician (SAR) was masked during the analysis. The optimal number of groups for each growth measure was selected on the basis of the best model fit using the Bayesian Information Criterion, and the number of infant in the smallest group that comprised at least 2·5% of the total sample. Every infant received a posterior probability of being in each group and was then assigned to the definitive group with the highest probability (appendix p 9).

Abdominal circumference growth trajectories comprised the primary independent variables. The trajectory tracking the 50th centile of the INTERGROWTH-21st standard^13^ was the reference group. Outcomes from the neurodevelopmental assessment were based on normative INTER-NDA scores.^17^, ^24^ Categories of low visual acuity (logarithm of the Minimum Angle of Resolution [logMAR] >0·4) and high contrast sensitivity (>3%) were based on Cardiff normal values.^25^

We used linear regression models to assess the relationships with continuous and normally distributed measures of growth, cognition, language, and fine motor and gross motor domains stratified by the duration of any breastfeeding (<7 months or ≥7 months). The positive affect domain was reverse coded, and both positive and negative scales were modelled as count data using generalised linear models, with a Poisson distribution and log-link function and a variance correction for over-dispersion. Vision outcomes were modelled as binary variables using Poisson regression with robust standard errors because they were not rare outcomes and negative binomial regression models often did not converge. The results represent a change in score or relative risk for each trajectory compared with the reference median growth trajectory. A p value of less than 0·05 was deemed statistically significant. We corrected our results for multiple comparisons using the Benjamini-Hochberg False Discovery Rate correction to control for type 1 error rate at <0·05.

We selected covariates suspected a priori to be in the causal pathway, using separate directed acyclic graphs for growth and neurodevelopmental outcomes (appendix p 21). In all adjusted models, we included the infant's sex and age at assessment, preterm birth, maternal age, maternal education, and smoking during pregnancy. As done previously,^26^ we conducted a primary stratified analysis to evaluate the effect modification by the duration of breastfeeding (<7 months or ≥7 months). We included abdominal circumference and head circumference trajectories in the models to explore the independent effect of abdominal circumference phenotypes.^10^

All analyses were performed using R (version 3.6.0; The R Core Team, Vienna, Austria) and STATA (version 15.1; StataCorp, College Station, TX, USA).

### Role of the funding source

The funder of the study had no role in study design, data collection, data analysis, data interpretation, or writing of the report.

## Results

Of the 5301 women screened, we enrolled 3598 between Feb 8, 2012, and Nov 30, 2019. Of these, 3206 had three or more scans between 14 and 37 weeks’ gestation (mean, 4·7 scans) and thus were included in the study. Each site's contribution to the total sample were: 397 (12·4%) of 3206 from Pelotas (Brazil), 647 (20·2%) from Oxford (UK), 554 (17·3%) from Soweto (South Africa), 502 (15·7%) from Karachi (Pakistan), 530 (16·5%) from Mae Sot (Thailand), and 576 (18·0%) from Nairobi (Kenya). There were 24 stillbirths or terminations resulting in 3182 eligible for birth outcomes. There were 21 deaths and 279 children excluded as they were older than 1 year when the neurodevelopmental assessments began, resulting in 2882 eligible for the 1 year assessment. There were 439 losses to follow-up, resulting in 2443 children assessed at 1 year (85% of those eligible). At the 2 year follow-up there were three additional deaths, nine missing all year outcomes and 446 lost to follow-up. However, 198 children who attended the 2-year visit did not attend the 1-year visit, leaving 2886 children eligible for the 2 year assessment. Overall, 2183 children completed the 2-year follow-up (76% of those eligible; appendix p 20).

**Figure 1:**
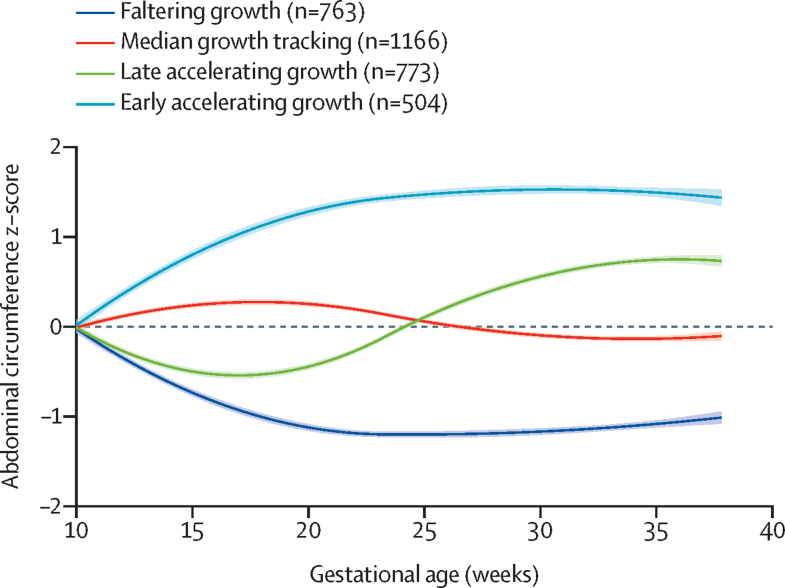
Fetal abdominal circumference growth phenotypes in the INTERBIO-21st fetal study (n=3206) Shaded bands represent 95% CIs for the splines used to summarise group trajectories.

The best fitting model of abdominal circumference growth had four trajectories (figure 1). The reference group was the median growth tracking (n=1166) phenotype with a growth pattern close to the 50th centile of the INTERGROWTH-21st standard.^13^ The faltering growth (n=763) phenotype showed abdominal circumference growth decreasing from early pregnancy to 20–23 weeks’ gestation; thereafter, the trajectory stayed at slightly less than –1 SD of the standard. The late accelerating growth (n=773) phenotype showed accelerating growth from 25 weeks’ gestation that was slightly less than +1 SD of the standard by 35 weeks’ gestation. The early accelerating growth (n=504) phenotype showed a rapid early growth that reached +1·5 SD of the standard by 20–25 weeks’ gestation and thereafter mirrored the faltering growth phenotype (figure 1).

**Figure 2:**
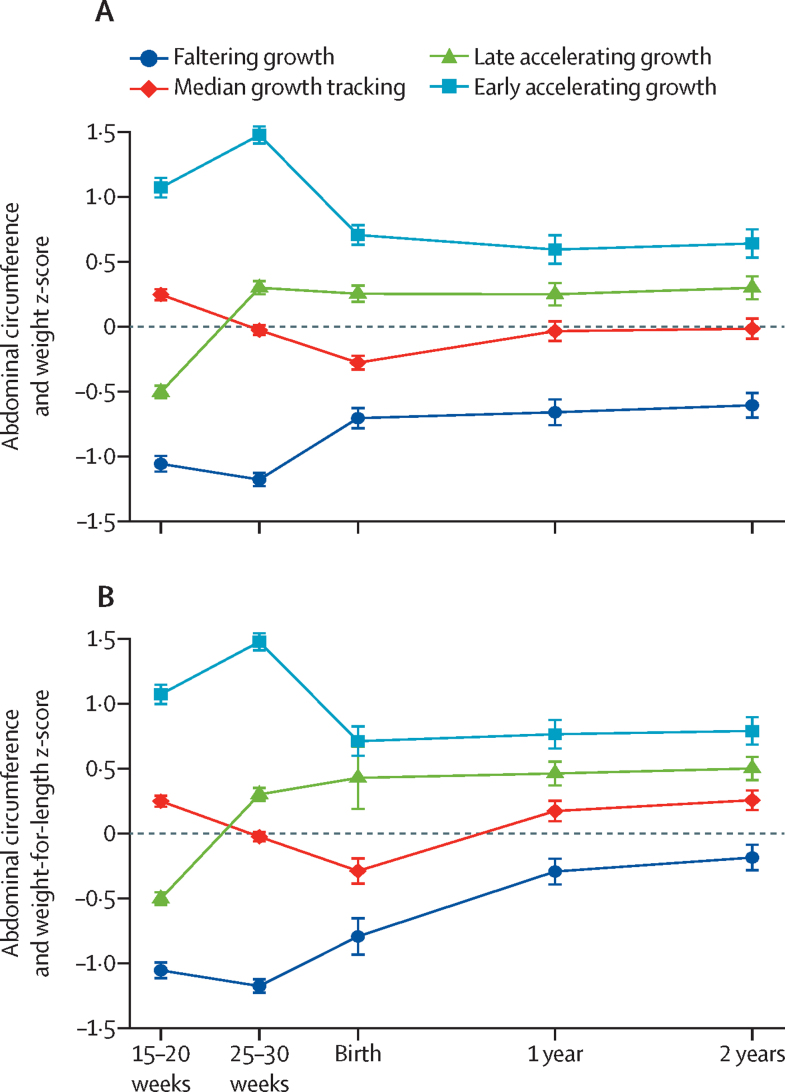
Growth z-scores for abdominal circumference during pregnancy, and weight (A) and weight-for-length (B) at birth and 1 and 2 years in the INTERBIO-21st fetal study Measurements up until birth (15–20 weeks’ gestation and 25–30 weeks’ gestation) were of abdominal circumference, and subsequently were either of weight (A) or weight-for-length (B). Fetal measurements of abdominal circumference were from the ultrasound scans. Error bars represent the 95% CIs of the means.

For each abdominal circumference phenotype we assessed the following measures: (1) abdominal circumference measured between the gestational age of 15–20 weeks and 25–30 weeks; (2) birthweight for gestational age and sex expressed as a z score of the INTERGROWTH-21st standard;^13^, ^15^ and (3) weight for age and sex and weight-for-length for age and sex at age 1 and 2 years, all expressed as z-scores of the WHO Child Growth Standards^16^ (figure 2; appendix pp 10–11).

Overall, from the second trimester of pregnancy to age 2 years, the faltering growth and early accelerating growth phenotypes were separated by the median growth tracking and late accelerating growth phenotypes (figure 2A). A similar pattern was observed for weight-for-length at ages 1 and 2 years, as a measure of early childhood adiposity (figure 2B); however, there was a slight upwards shift across the four phenotypes, indicative of a greater increase in weight attainment in proportion to height, particularly for the faltering growth phenotype. Ultrasound-derived estimated fetal weight showed almost identical patterns to both weight and weight-for-length at age 1 and 2 years (data not shown).

Mothers of children in the faltering growth phenotype tended to be shorter and less educated, had lower pre-pregnancy BMI, and a higher rate of smoking and preeclampsia (appendix p 16). The mothers of children in the late accelerating growth and early accelerating growth phenotypes tended to have overweight or obesity, and have gestational diabetes and pregnancy-induced hypertension (appendix p 15).

The median growth tracking and faltering growth phenotypes had a similar mean (SD) duration of breastfeeding: 13·1 (8·8) months for median growth tracking versus 13·9 (8·8) months for faltering growth (p=0·08). The early accelerating growth (11·1 [8·4] months) and late accelerating growth (12·6 [8·9] months) phenotypes had slightly shorter durations than the median growth tracking phenotype (p=0·002). Breastfeeding for 7 months or more increased the postnatal growth patterns of weight, head circumference, and weight-for-length for the faltering growth phenotype, without statistically significant interactions between the two breastfeeding strata. For the late accelerating growth and early accelerating growth phenotypes, children breastfed for 7 months or more had significantly higher weight, larger head circumference, and greater weight-for-length than the median growth tracking phenotype; however, the interaction terms by breastfeeding duration were not significant (appendix pp 13,17,22).

Finally, we assessed the association between the abdominal circumference phenotypes and neurodevelopmental outcomes at 2 years, stratified by breastfeeding duration (appendix pp 13,17). The early accelerating growth phenotype scored highest on the language and positive affect domains of the INTER-NDA,^24^ only if infants were breastfed for 7 months or more. The early accelerating growth phenotype showed significantly lower risk for poor vision outcomes at 2 years than the median growth tracking phenotype, as expressed by low LogMAR for visual acuity and low percentage for contrast sensitivity,^25^ regardless of breastfeeding duration. Conversely, the faltering growth phenotype had the highest risk for poor visual outcomes, but the risk for low acuity (high LogMAR scores) was reduced among infants breastfed for 7 months or more (appendix pp 14–15,18–19).

There were 26 significant findings with abdominal circumference growth trajectories related to 2-year outcomes, of which 24 (92%) were significant after correcting for multiple comparisons; of these 24 significant findings, nine were for late accelerating growth phenotypes and 10 were for early accelerating growth phenotypes, as well as five of the seven significant findings for the faltering growth phenotype.

**Figure 3:**
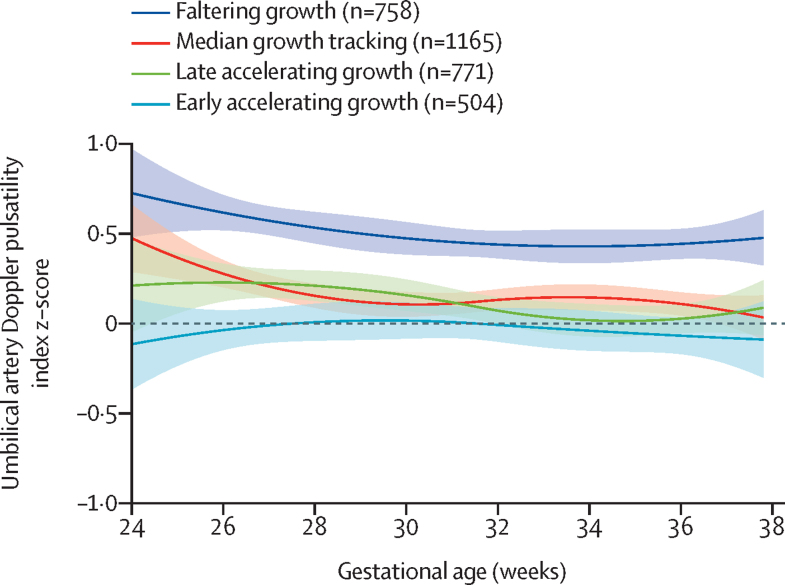
Umbilical artery Doppler Pulsatility Index trajectories in the INTERBIO-21st fetal study N=3206. Group membership was established by an analysis of fetal abdominal circumference growth trajectories. Shaded bands represent the 95% CIs for splines used to summarise group trajectories.

We modelled the mean umbilical artery Doppler Pulsatility Index values for each phenotype, expressed as z-scores of the international standard.^14^ From 24 weeks’ gestation onwards, Doppler Pulsatility Index trajectories for the faltering growth phenotype were consistently higher than for the other three phenotypes, whose 95% CIs overlapped during the third trimester (figure 3).

**Figure 4:**
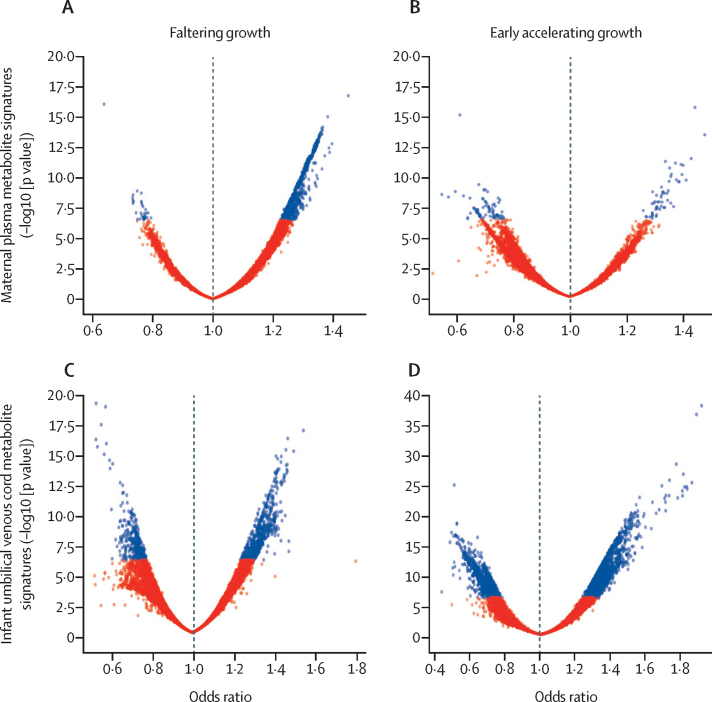
Volcano plot of odds ratio distributions for each maternal metabolite according to the faltering growth and early accelerating growth phenotypes Volcano plots of odds ratios and p-values, adjusted by maternal age and newborn sex, from maternal plasma (Aand B) and linked umbilical venous cord (C and D) metabolite signatures for the faltering growth and early accelerating growth phenotypes of ultrasound-based fetal abdominal circumference growth trajectories (n=2713 women and n=2430 newborn babies). Blue dots indicate p<10^−6^.

Few maternal or umbilical cord venous metabolites among the median growth tracking and late accelerating growth phenotypes reached the strict criterion for statistical significance (p<10^−6^) and, as a result, we decided to focus our efforts on understanding the metabolic differences between the most divergent growth trajectories (figure 1); these two groups displayed more statistically significant signatures when compared with the other groups. Figure 4 presents volcano plots of the odds ratio (OR) distributions for each maternal metabolite according to the faltering growth and early accelerating growth phenotypes estimated from logistic regression analysis, adjusted for maternal age and newborn sex. These phenotypes were associated with 709 statistically significant maternal metabolites with positive effect for the faltering growth phenotype and 54 metabolites (appendix pp 28–43) for the early accelerating growth phenotype, and 31 statistically significant maternal metabolites with negative effect for the faltering growth phenotype and 76 metabolites (appendix pp 43–45) for the early accelerating growth phenotype (figure 4A and B). 33 metabolites (appendix p 46) overlapped between the faltering growth and early accelerating growth phenotypes, which were also associated with 627 (for the faltering growth phenotype) and 1675 (for the early accelerating growth phenotype) statistically significant umbilical cord venous metabolites with positive effect. These metabolites were also associated with 364 (for the faltering growth phenotype) and 1225 (for the early accelerating growth phenotype) with negative effect (figure 4C and D), with 604 overlapping metabolites (appendix pp 46–59).

We visualised how metabolites associated with these two phenotypes diverged at the metabolome-wide level using dimensional reduction with supervised Uniform Manifold Approximation and Projection (appendix p 5),^18^ including all detectable molecules. The data separated into two clusters, indicating distinctive underlying metabolite abundance profiles between the faltering growth and early accelerating growth phenotypes in both maternal and umbilical cord venous plasma (appendix p 24).

**Figure 5:**
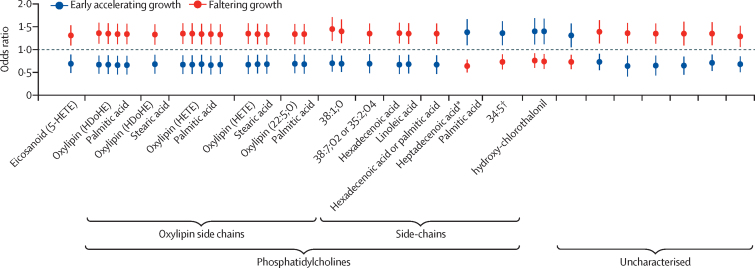
Odds ratios for the 20 unique molecules identified that overlapped between the faltering growth and early accelerating growth phenotypes Association between early pregnancy, overlapping maternal metabolite signatures, and the faltering growth and early accelerating growth phenotypes of ultrasound-based, fetal abdominal circumference growth trajectories in the INTERBIO-21st fetal study (n=2713). Data are presented as odds ratios and 95% CIs. Models are adjusted for maternal age and fetal sex. *Or pentadecylic acid or oleic acid. †Putative.

Figure 5 presents ORs (95% CI) for the 20 unique molecules (p<10^−6^ identified that overlapped the faltering growth and early accelerating growth phenotypes for which we performed compound identification. Grouped ORs (95% CI) represent unique molecules, either multiple isotopes or adducts. Most metabolites associated with the faltering growth phenotype had ORs close to 1·5, indicative of an upregulation of metabolic pathways associated with impaired fetal growth; conversely, these metabolites had a reciprocal relationship with the early accelerating growth phenotype, which suggests that the same pathways are downregulated when fetal growth is accelerated.

Among the 20 unique metabolite annotations overlapping the faltering growth and early accelerating growth phenotypes, seven were uncharacterised molecules; one was an eicosanoid (5-HETE) and 11 were phosphatidylcholines (five with oxylipin and six with palmitic acid as the most common sidechains). We also identified hydroxy-chlorothalonil, a metabolite of the fungicide, chlorothalonil, in a positive association with the early accelerating growth phenotype (figure 5).

We selected the top ten metabolite annotations for each positively and negatively associated with the faltering growth and early accelerating growth phenotypes (n=40); of these, 30 (faltering growth=14 and early accelerating growth=16) were distinct from the 33 metabolite annotations that overlapped the two phenotypes. Among the 14 that were exclusive to the faltering growth phenotype, an additional five phosphatidylcholines were identified. Of these, four had a positive association with the faltering growth phenotype and contained oxylipin or the oxylipin precursors docosahexaenoic acid and hydroperoxylinoleic acid as a sidechain, along with saturated fatty acids (eg, stearic or palmitic acid) as the other sidechain. The fifth phosphatidylcholine, with a saturated fatty acid sidechain (1-decanoyl-2-heneicosanoyl-sn-glycero-3-phosphocholine), had a negative association with the faltering growth phenotype. Additionally, threonine (or homoserine), the precursor of the amino acids glycine and serine, was putatively identified as positively associated with the faltering growth phenotype. All 16 metabolite annotations that were exclusive to the early accelerating growth phenotype were not identified.

We compared metabolites identified in both the maternal and umbilical cord venous samples. In the faltering growth phenotype, 603 molecules were associated with both sample sets, of which 47 were significant (p=8·3 × 10^−5^). In the early accelerating growth phenotype, 107 molecules were associated with both sample sets, of which 31 were significant (p=4·6 × 10^−4^). However, only two metabolites overlapped the faltering growth and early accelerating growth phenotypes in the maternal and umbilical cord venous cord samples, and these uncharacterised metabolites were positively associated with the faltering growth phenotype. Hydroxyl-chlorothalonil was also observed in cord blood samples, but was not significantly associated with any growth trajectory (data not shown).

## Discussion

In this primary analysis of the INTERBIO-21st fetal study, we present longitudinal abdominal circumference phenotypes associated with different patterns of feto–placental blood flow (reflecting the varied placental transfer of essential nutrients); birthweight; postnatal weight, weight-for-length, and visual and neurodevelopmental scores up to age 2 years; and maternal and newborn baby metabolite signatures.

Abdominal circumference was evaluated as the main independent variable principally because it approximates the growth of the fetal liver and abdominal subcutaneous fat. Hepatic volume in uncomplicated pregnancies increases 10 times from 20 to 36 weeks’ gestation; in growth-restricted fetuses, the fall in z-scores for abdominal circumference and hepatic volume is similar.^27^ Thus, we identified, across a key period of human development, organ-specific novel phenotypes or so-called exposotypes.^28^ This concept is central to our efforts to create a functional classification of fetal growth alterations with substantial effects during childhood, rather than mere biometrically based descriptions.

Our findings, based on a systems biology approach, add to the existing evidence that understanding the influence of the maternal exposome on human development from early pregnancy onwards requires much deeper phenotyping to complement syndromic classifications, such as preterm birth.^9^, ^12^ Our approach has identified not only a strong association between distinct trajectories of fetal growth and childhood outcomes, but also the putative metabolic pathways that cause the biological mechanisms.

In addition, one of our key observations, with considerable biological and programmatic implications, is the existence of an important developmental window at 20–25 weeks’ gestation. During this period, fetal growth patterns emerge that appear to be organ-specific: after an initial period of accelerated or deaccelerated growth, abdominal circumference measures start to stabilise, just as head circumference growth starts accelerating or deaccelerating (appendix p 27). We acknowledge that we did not find a significant interaction between breastfeeding and postnatal growth in the context of this early pregnancy effect; however, understanding the biological implications of these interactions is the aim of our ongoing analyses.

The faltering growth phenotype, in particular, showed early growth restriction and reduced feto–placental blood flow throughout pregnancy, without postnatal catch-up growth. These observations and the maternal characteristics of mothers of children in the faltering growth phenotype suggest that fetuses and children that follow the faltering growth pattern are not simply constitutionally small, and there is a faltering growth phenotype subgroup, associated with preeclampsia and reduced umbilical artery flow, that manifests early in pregnancy. Conversely, the late accelerating growth and early accelerating growth phenotypes were associated with the metabolic syndrome, overweight and obesity, gestational diabetes, or pregnancy-induced hypertension).

Hence, the variability seen in fetal hepatic growth and subcutaneous fat deposition needed for postnatal thermoregulation as seen in figure 1, is related to specific risk and exposome factors that persist in affecting growth up to 2 years. In infants with the faltering growth phenotype, postnatal adiposity steadily increased, as seen in figure 2B, mostly because of a slower growth in length rather than a faster increase in weight. It is likely, therefore, that an adverse maternal exposome early in pregnancy, when skeletal growth velocity is at its highest,^29^ has a lasting effect on infant length and height and adiposity, which might also explain why a mismatch between faltering abdominal circumference growth and rapid postnatal weight gain is associated with elevated insulin resistance, fat accumulation, and high blood pressure in early childhood.^30^

Our metabolomic analyses show that few maternal metabolites are associated with abdominal circumference trajectories that diverge early in pregnancy. The metabolite hydroxy-chlorothalonil in maternal plasma had a significant reciprocal association with the two extreme abdominal circumference phenotypes, as shown in figure 5. This association was similar in the umbilical cord venous samples (although not significantly), supporting the placental transfer of chlorothalonil as previously reported.^31^ However, the mechanism by which this fungicide, which was banned in Europe in 2020, might stimulate fetal growth is unclear.

Five (oxylipin [HDoHE] palmitic acid, oxylipin [HDoHE] stearic acid, oxylipin [HETE] palmitic acid, oxylipin [HETE] stearic acid, and oxylipin [22:5;0] palmitic acid) of the 11 overlapping phosphatidylcholines between the faltering growth and early accelerating growth phenotypes, as shown in figure 5, and four of those exclusively related to the faltering growth phenotype (PC [36:4;O3], PC [18:0/20:4;O2] stearic acid or DiHETE, PC [16:0/22:6] palmitic acid, and PC [16:0/18:2;O2] palmitic acid or hydroperoxylinoleic acid), had either saturated fatty acids or oxylipin as sidechains. Of the four phosphatidylcholines that were overlapping but with a higher abundance in the faltering growth phenotype, three (PC [38:7;O2] or PC(35:2;O4), hexadecenoic acid or linoleic acid, and hexadecenoic acid or arachidonic acid or palmitic acid) had a palmitic acid or palmitoleic acid sidechain, or pentacosanoic acid sidechain, a very long-chain saturated fatty acid; the fourth phosphatidylcholine (38:1;O) was linked to a sphingolipid, which has been associated with cardiovascular events and SGA.^32^, ^33^ Of the two phosphatidylcholines with a higher abundance in the early accelerating growth phenotype, one had the saturated heptadecenoic acid (margaric acid) and the other a long-chain fatty acid (putative 34:5).

We speculate that an explanation for the reciprocal association of oxylipins linked to phosphatidylcholines is that they function as endogenous signalling molecules, serving as ligands for nutrient-sensing peroxisome proliferator-activated receptors (PPARs).^34^ All three PPAR isotypes are highly expressed in placental tissue throughout pregnancy and play a key role in trophoblast differentiation and regulating energy metabolism, organ growth, and adipose deposition in the fetus.^35^ There is in vitro evidence that the activation of PPARs alters placental function by downregulating 11β-hydroxysteroid dehydrogenase type 2 expression.^36^ Placental 11β-hydroxysteroid dehydrogenase type 2 reduces the deleterious effects of fetal exposure to maternal glucocorticoids, and reduced expression of this enzyme is associated with intrauterine growth restriction.^37^

Alternatively, oxylipins, which are a broad range of oxidised lipids derived from polyunsaturated fats, might be affecting placental function via inflammation and oxidative stress.^38^ Likewise, saturated fatty acids might affect placental function because they induce trophoblast lipoapoptosis in vitro, a cause of programmed cell death.^39^ Thus, the balance between saturated fatty acids and polyunsaturated fatty acid phosphatidylcholine sidechains might establish the trajectory of early fetal growth.

Conclusions drawn from metabolomic studies about the specific effects of oxylipins and phosphatidylcholines in pregnancy, most of which are dependent on maternal diet, are speculative given the heterogeneity of these compounds and the complexity of lipid biology. Summarising this literature is also difficult because multiple pregnancy outcomes have been explored, and there are well recognised limitations to metabolomic studies, including the low reproducibility of results and inconsistent reporting practices, precluding formal meta-analysis.^40^

An initial inspection of the results from our formal systematic review of metabolomic studies in pregnancy,^41^ which builds on a previous review,^42^ has identified 16 unique studies presenting data on metabolomic markers of lipid metabolism associated with SGA, intrauterine growth restriction, and fetal growth restriction; only four collected maternal blood samples early in pregnancy. The SCOPE cohort study found glycerophospholipids, including phosphatidylcholines, to be associated with SGA.^33^, ^43^ Welch and colleagues^44^ identified eicosanoids to be positively associated with SGA, some of which were negatively associated with large for gestational age (LGA). Sovio and colleagues^45^, ^46^ showed that a ratio of two positively and two negatively associated metabolites predicted intrauterine growth restriction at term, as well as LGA, and that it is inversely associated with birthweight. Finally, McBride and colleagues^47^ reported an association between different alterations in lipid biology and SGA; however, they used targeted nuclear magnetic resonance, which identifies fewer metabolites. McBride and colleagues^48^ also analysed samples taken later in pregnancy (26–28 weeks’ gestation), and showed that 4-hydroxyglutamate and glycerol combined with maternal risk factors improved the prediction of SGA and LGA.

In summary, this well powered, primary analysis of the INTERBIO-21st fetal study provides uniquely robust information because we followed a large, prospective cohort from low-income, middle-income, and high-income countries at moderate or high risk of pregnancy and postnatal complications from early pregnancy to age 2 years; all pregnancies were accurately dated by ultrasonography and early infant feeding was human-milk based. Dedicated research staff obtained ultrasound and postnatal standard anthropometric measures using the same equipment. We transformed all measures to z-scores of international standards, allowing multiple regression analyses to be conducted across time using the same conceptual framework. Finally, our large sample size and longitudinal study design allowed us to explore several variables in the causal pathway that are often matched in small case–control studies.

There are limitations intrinsic to statistical analysis covering such a range of variables and unit measures. Our phenotypic modelling methods could only approximate the abdominal circumference trajectories’ starting point early in pregnancy and these methods were specific to our cohort, although future studies could use the same methods. The visual representation in the appendix (p 24) needs to be interpreted with caution, avoiding over-interpretation, because the dimensionality reduction technique Uniform Manifold Approximation and Projection is not quantitative; the algorithm simply optimises the visualisation of the data separation using phenotypic labels. Most biomolecules were in low abundance but the metabolomic analyses were conducted using relative, rather than absolute, abundance concentrations (ie, qualitative differences). We might have missed other important metabolites because of selection bias occurring when isolating metabolites for measurement. We could not establish the precise polyunsaturated fatty acid derivation of the oxylipins identified, which particles the phosphatidylcholines were associated with, and the directionality of signalling (ie, to or from the placenta), which we believe is important because metabolites derived from essential fatty acids are well conserved. Nor was it possible to elucidate all the biological pathways involved because many molecules were uncharacterised. Of note, however, is that both positive and negative method data were collected, which expanded the pool of metabolites observed. Our four-trajectory model was data driven, based on criteria defined a priori. The faltering growth group could possibly be further subclassified^49^ according to the timing of deceleration, but this was problematic because group membership was specific to the model used and splitting the faltering growth group post-hoc would be inadvisable. Lastly, the use of middle cerebral artery Doppler to capture the so-called brain-sparing effect,^50^ in addition to umbilical artery Doppler as a measure of feto–placental blood flow, could have provided further characterisation of the groups.

We have presented results stratified by breastfeeding duration, which can introduce bias by treating potentially mediating variables as effect modifiers if faltering growth infants are breastfed for shorter periods; however, this was not the case in our cohort. Hence, we present both stratified and overall results because breastfeeding duration is known to be an effect modifier for infant growth and development, and more importantly, if confirmed, it has considerable relevance clinically and for public health generally.

Overall, our present and past results^13^ (appendix p 27) strongly support the existence of: (1) organ-specific patterns of fetal growth trajectories that follow distinct growth, adiposity, and visual and neurodevelopmental outcomes in early childhood; (2) the influence of breastfeeding on these patterns; (3) a crucial window at 20–25 weeks’ gestation when the growth of the fetal cranium and abdomen can accelerate or decelerate, resulting in sustained trajectories; and (4) a strong association between restricted abdominal circumference growth, reduced utero–placental blood flow, and the maternal exposome involving phospholipid metabolism, linked to postnatal growth trajectories.

## Data sharing

Anonymised data will be made available upon reasonable request for academic use and within the limitations of the informed consent. Requests should be made to the corresponding author. Every request will be reviewed by the INTERBIO-21st Consortium Executive Committee. After approval, the researcher will need to sign a data access agreement with the INTERBIO-21st Consortium.

## Declaration of interests

ATP is supported by the Oxford Partnership Comprehensive Biomedical Research Centre with funding from the National Institutes of Health Research Biomedical Research Centre funding scheme; and is a senior advisor of Intelligent Ultrasound. All other authors declare no competing interests.
