# Supplementary material for: Association between fetal abdominal growth trajectories, maternal metabolite signatures early in pregnancy, and childhood growth and adiposity: prospective observational multinational INTERBIO-21st fetal study
Source: Lancet Diabetes Endocrinol. 2022 Oct;10(10):710–9. doi: 10.1016/S2213-8587(22)00215-7 (PMC9622423; doi:10.1016/S2213-8587(22)00215-7)
Supplement: Supplementary appendix [file mmc1.pdf]

THE LANCET
Diabetes & Endocrinology

Supplementary appendix

This appendix formed part of the original submission and has been peer reviewed. We post it as supplied by the authors.

Supplement to: Villar J, Ochieng R, Gunier RB. Association between fetal abdominal growth trajectories, maternal metabolite signatures early in pregnancy, and childhood growth and adiposity: prospective observational multinational INTERBIO-21st fetal study. *Lancet Diabetes Endocrinol* 2022; published online Aug 25. https://doi.org/10.1016/S2213-8587(22)00215-7.

SUPPLEMENTARY APPENDIX

CONTENTS

**Methods**	**2**
Pregnancy data	2
Fetal ultrasound scans	2
Anthropometric measures	2
Infant follow-up	3
Neurodevelopment assessment	3
Clinical data management	3
Sample collection and processing	4
Metabolomic analysis	4
**Results**	**6**
Fetal phenotypic characterisation and postnatal follow-up	6
**References**	**8**
**Supplementary Tables 1-8**	**9**
**Supplementary Figures 1-7**	**20**
**Metabolomic data**	**28**
**INTERBIO-21st Committees and local investigators**	**60**

METHODS

Pregnancy data

A comprehensive set of demographic, clinical, pregnancy and postnatal growth and morbidity variables was obtained prospectively using an electronic data entry system (www.interbio21.org.uk). Baseline information included demographic and nutritional characteristics, medical, gynaecological and obstetric history and current pregnancy conditions. Pregnancy follow-up information included standard antenatal care variables, pregnancy complications and morbidities, use of supplements or medication, and referral to another level of care or hospital.

Fetal ultrasound scans

Fetal abdominal circumference (AC) measures, the primary marker to assess growth of the fetal abdominal organs and adipose tissue, were obtained in an axial plane, with the umbilical vein in the anterior third of the fetal abdomen (at the level of the portal sinus) and the stomach bubble visible. Both the ellipse facility and the two diameters method were used, placing the calipers on the outer border to capture subcutaneous fat. All measurements were taken three times from three separately generated images. To avoid expected-value bias,[1] the ultrasound machines were adapted to ensure the ultrasonographers were "blinded" to the actual values, which were transferred electronically and only available for clinical use, after the examination was completed. Gestational age-specific z-scores were estimated using the INTERGROWTH-21st Fetal Growth Standard.[2] Eleven fetal AC measures with z-scores >5 or < -5 were excluded from the analysis.

Detailed documentation on measurement acquisition protocols, the unique standardisation procedures, data-collection forms, and electronic data transfer strategies are available at www.interbio21.org.uk.

Anthropometric measures

A first trimester maternal body mass index (BMI) was calculated and categorised as normal weight (BMI ≥18.50 kg/m^2 to ≤24.99 kg/m^2), overweight (BMI ≥25.00 kg/m^2 to ≤29.99 kg/m^2) or obese (BMI >30 kg/m^2), according to World Health Organization (WHO) definitions.[3]

Newborn measures were obtained within 12 h of birth (and no later than 24 h), using the same equipment at all sites: electronic scale (Seca, Hamburg, Germany) for birth weight (sensitivity of 10 g up to 20 kg) and a specially designed Harpenden infantometer (Chasmors Ltd, London, UK) for recumbent length. Head circumference was measured using a metallic non-extendable tape (Chasmors Ltd, London, UK).

All lead anthropometrists were standardised at regular intervals using training materials based on WHO protocols. Two anthropometrists took all newborn measures twice

independently and compared their values with the maximum allowable differences: newborn weight, 50 g; length, 7 mm; and head circumference, 5 mm. If the difference between the two measures exceeded these values then both observers independently repeated that measurement a second, and if necessary, a third time.[4]

Across all sites, standardised clinical care and feeding practices were implemented. (www.intergrowth21.org.uk). Exclusive breastfeeding up to 6 months and appropriate nutritional support for infants born preterm were promoted during and after pregnancy as recommended.[5]

Infant follow-up

At age 1 and 2, detailed information was obtained from the mother about the infant's health, severe morbidities, hospitalisations, duration of breastfeeding, timing of the introduction of solid food, age at weaning, feeding practices and food intake, using specially produced forms (www.intergrowth21.org.uk).[6,7]

Neurodevelopment assessment

The INTER-NDA includes 37 culture-specific items measuring six neurodevelopmental domains - cognition, language (expressive and receptive), fine and gross motor skills, and positive and negative behaviour - in an assessment time of 15 min on average. The tool has been validated against the Bayley Scales of Infant Development III edition, showing good to moderate agreement,[8] and good levels of inter-rater (k=0.70; 95% CI: 0.47–0.88) and test/re-test reliability (k=0.79; 95%CI: 0.48–0.96).[9]

Attentional problems and emotional reactivity were measured on the respective subscales of the Preschool Child Behavior Checklist (CBCL);[10] responses were based on caregiver reports. Vision was assessed using the Cardiff Visual Acuity and Contrast Sensitivity tests for binocular vision (Supplementary Table 2).[11] All INTER-NDA assessors were subject to a protocol adherence and reliability assessment following training, only those with protocol adherence scores in excess of 90% and inter-rater reliability of >0.8 conducted assessments. Normative standards were developed following WHO guidelines.[12]

Clinical data management

Demographic, and all maternal and newborn clinical data, were collected initially on paper forms and then entered locally into an on-line data management system, developed for the INTERGROWTH-21st Project (MedSciNet, London, UK), which sits on a secure MedSciNet server. Blinded data from the ultrasound machines were transferred directly to the database in Oxford. The anonymised, de-identified databases are only accessible to designated personnel, including the Bill & Melinda Gates Foundation as part of a data sharing agreement. Users from each study site only have access at present to their own data; a

small number of global administrators have access to all the data on a high security, encrypted server.

Sample collection and processing

Early pregnancy maternal and linked umbilical cord venous plasma samples were obtained as previously described,[13] stored at -80°C and transported on dry ice to the central laboratory (Sapient Bioanalytics, San Diego, CA, USA).

Samples were thawed overnight at 4°C and placed on an orbital shaker at 550 rpm at 4°C for 10 min. Twenty microlitres of sample were transferred to a shallow 96-well microtitre plate containing 80 microlitres of extraction solution [MeOH with 0.5% acetic acid, with the following internal standards: $^{13}C_5{}^{15}N_2$-Glutamine (Sigma-Aldrich), MAPCHO-12-d38 (Avanti Lipids),$^{13}C_5{}^{15}N_1$-Glutamate (Sigma-Aldrich), CUDA (Cayman Chemicals)]. Samples were shaken at 550 rpm at 4°C for 10 min followed by centrifugation at 6000g at 4°C for 10 min. Supernatant was then transferred to a 384-well polypropylene plate containing 35:65 or 75:25 methanol:water (for pos/neg mode analysis). All samples were de-identified prior to receipt and all analyses were completed in a blinded fashion.

Metabolomic analysis

Samples were analysed by liquid chromatography-mass spectrometry (LC-MS) using a modified Agilent RapidFire 360 sample injector coupled to a high-resolution Agilent 6545B QToF mass spectrometer (Agilent Technologies, Santa Clara, CA, USA). Samples were injected onto a custom packed, reverse phase column for data acquisition in both positive and negative ionisation modes using mobile phases consisting of water, methanol and acetonitrile. A separate aliquot of pooled commercial plasma (BioIVT, Westbury, NY, USA) was also extracted and injected as an external QC sample. The mass spectrometer parameters were set as follows: dry gas temp of 365 °C, dry gas flow rate of 13 L/min, nebuliser gas of 60 psi, sheath gas temp of 400 °C, sheath gas flow rate of 12 L/min, source voltage of (+)3500 / (-) 3000 V and nozzle voltage of (+) 50 / (-) 1000 V, mass range set to 50 – 1700 m/z, and data were collected at 8 spectra/s.

Rigorous data QC was performed using the panel of isotopically labeled internal standards and interval pooled plasma samples to monitor fluctuations in extraction efficiency, instrument sensitivity, matrix artifact and mass accuracy. For system suitability, mass calibration was performed prior to each 384-well plate run and used to assess mass accuracy, mass resolution, detector sensitivity and instrument cleanliness. For each 384-well plate run, the QC was as follows: isotopically labeled internal standards were added to each plasma sample at the first preparation step to monitor matrix effects; bulk pre-aliquoted commercial pooled plasma (BioIVT) was placed in wells A1, D12 and H12 of each 96-well plate and prepared identically to samples (internal bracket QC sample); bulk pre-aliquoted

commercial pooled plasma was prepared external to the 96-well plate by hand (external bracket QC sample), and a preparation blank was prepared during each 384-well plate to assess background. Overall coefficient of variation (%CV) was monitored for internal standards in the samples. For samples with %CV >25% and total ion current measures >3 standard deviations (SD) from the mean, samples were re-injected and removed from the final analysis if found to exhibit significant matrix suppression (164/2,881, 5.6% total samples).

A total of 2,713/3,206 (84.6%) maternal and 2,430/3,206 (75.8%) umbilical cord plasma samples passed QC with 67,575 rLC-MS derived metabolite features (34,399 negative ionisation mode and 33,176 positive ionisation mode) and were used in the final analyses. Raw data were converted to mzXML using MSConvert (Proteowizard 3.0).[14] Chromatographic drift was then assessed and corrected based on common landmarks observed in all samples.[15] Metabolite features were then extracted from drift-corrected mzXML files using custom imaging processing-based software.[16] Following data extraction molecular features were subsequently normalised to account for plate-to-plate variation using a batch median normalisation metric with correction for median levels.

We conducted logistic regression analysis to identify metabolite features associated with the AC phenotypes, controlling for maternal age and fetal sex, with separate models for each metabolite feature. To minimise the influence of extreme values in the regression models, the abundance of upper outlier metabolite features was capped at the 99th centile level of that metabolite. Missing metabolite feature abundances (those below the limit of detection) were imputed using the minimum detected abundance per metabolite because these samples did not necessarily have zero abundance but were likely to have an actual signal below the detection limit. The median (IQR) percentage of samples with metabolite features that were below the limits of detection and imputed with the minimum detected abundance was 0.9% (0 - 10%). We imputed missing data on maternal age using the median value.

Metabolite features were standardised by subtracting the mean and scaling to unit variance with the standard score of metabolite features calculated as $z=(x - u)/s$, where u is the mean and s is the SD of the metabolite abundances. A single metabolome-wide significance threshold of $p < 1x10^{-6}$ was used to identify the principal associated metabolite features accounting for multiple comparisons and the lack of independence among metabolites. We determined this significance level using an estimate of the independent number of metabolite features in a typical dataset to account for multiple comparisons and the minimal p values were determined from permutation analysis on phenotypes across studies (Supplementary Fig. 6). Thresholds for investigating overlaps between datasets were established based on the number of metabolites examined. All statistical analyses were performed blinded to the identity of the four AC phenotypes. The output is the odds ratio

(OR) for the metabolite; a fetal AC growth trajectory relationship > 1 indicates the metabolite abundance was higher in the group and < 1 indicates lower abundance.

To visualise the metabolite feature data, we employed supervised Uniform Manifold Approximation and Projection (UMAP) using the categorical label information from the fetal AC phenotypes for supervised dimension reduction (neighbours=50, minimum distance=0.1) with the Python package UMAP-learn.[17]

RESULTS

Fetal phenotypic characterisation and postnatal follow-up

The probability of correctly classifying each group was much higher than the probability of being included in another group (average posterior probabilities 0.77-0.89; odds of correct classification 4.9-22.3) indicating minimal classification error (Supplementary Table 1).

We explored the contribution of study sites to the 763 fetuses included in the FG and EAG phenotypes. There was a very strong effect of socio-economic health care conditions (independent of ethnic origin) to the proportional contribution of fetuses in the FG phenotype, ranging from 5.1% and 4.0% from the cities of Pelotas, Brazil and Oxford, UK; through 14.9% and 15.7% from the selected areas of Karachi, Pakistan and Nairobi, Kenya (both sites belong to very similar socio-cultural and health care systems) to 31.4% and 28.7% from rural Mae Sot, Thailand and Soweto, South Africa, the last two sites at highest risk of intrauterine growth restriction (IUGR). The same pattern, in the opposite direction, was seen for the EAG phenotype. Hence, we did not adjust for study site because the related environmental factors were likely to be aetiologically associated with growth patterns.

Stratified trajectories for the FG and EAG phenotypes across four strata of study sites (with Oxford and Pelotas considered separately) were estimated to explore the AC growth trajectories of fetuses within these four strata. There were similar trajectory patterns among strata with the expected variability at the end of pregnancy due to sample size variations (Supplementary Fig. 5).

We estimated beta coefficients (95% CI) for the AC phenotypes as the independent variable, using the MGT phenotype as the reference group. We adjusted for maternal education and age, preterm birth, smoking, and infant sex and age at assessment using the child's four anthropometric measures at age 2 as dependent variables, expressed as z-scores of the WHO Child Growth Standards. The longitudinal trajectories of AC growth are presented relative to the standards as deviations from the expected gestational-age specific z scores. The AC trajectories were adjusted for the fetal head circumference (HC) trajectories during the same gestational age period; they can be interpreted as AC trajectories conditional on

HC changes over the same period. For all four anthropometric measures, the FG phenotype remained significantly below the MGT phenotype, whilst the EAG phenotype was statistically significantly above the reference group.

The FG neonates were more likely to be admitted to a NICU and be small for gestational age with low birth weight (Supplemental Table 3). The EAG and LAG phenotype mothers were the tallest and heaviest: 18% were obese (BMI>30 kg/m^2) and over 50% had a BMI >25 kg/m^2. Pregnancy-induced hypertension was more common in the EAG and LAG phenotypes. Conversely, the FG phenotype mothers had a slightly higher rate of preeclampsia. They also tended to be younger, more likely to be smokers, and less likely to be married, have a university education, or a history of thyroid or another endocrine disease than other mothers (Supplementary Table 6).

REFERENCES

1. Drukker L, Droste R, Chatelain P, Noble JA, Papageorghiou AT. Expected-value bias in routine third-trimester growth scans. Ultrasound Obstet Gynecol 2020; 55(3): 375-82.
2. Papageorghiou AT, Ohuma EO, Altman DG, et al. International standards for fetal growth based on serial ultrasound measurements: the Fetal Growth Longitudinal Study of the INTERGROWTH-21st Project. Lancet 2014; 384(9946): 869-79.
3. World Health Organization (WHO). BMI classification. 2006. http://apps.who.int/bmi/index.jsp?introPage=intro_3.html&2014).
4. Cheikh Ismail L, Knight H, Ohuma E, et al. Anthropometric standardisation and quality control protocols for the construction of new, international, fetal and newborn growth standards: the INTERGROWTH-21st Project. BJOG 2013; 120 Suppl 2: 48-55.
5. Cheikh Ismail L, Giuliani F, Bhat BA, et al. Preterm feeding recommendations are achievable in large-scale research studies. BMC Nutr 2016; 2(9).
6. Dewey KG, Cohen RJ, Arimond M, Ruel MT. Developing and Validating Simple Indicators of Complementary Food Intake and Nutrient Density for Breastfed Children in Developing Countries. Final Report. Washington, D.C.: Academy for Educational Development (AED), 2005.
7. WHO Multicentre Growth Reference Study Group. Complementary feeding in the WHO Multicentre Growth Reference Study. Acta Paediatr Suppl 2006; 450: 27-37.
8. Murray E, Fernandes M, Newton CRJ, et al. Evaluation of the INTERGROWTH-21st Neurodevelopment Assessment (INTER-NDA) in 2 year-old children. PLoS One 2018; 13(2): e0193406.
9. Fernandes M, Stein A, Newton CR, et al. The INTERGROWTH-21st Project Neurodevelopment Package: A novel method for the multi-dimensional assessment of neurodevelopment in pre-school age children. PLoS One 2014; 9(11): e113360.
10. Achenbach TM, Rescorla LA. Manual for the ASEBA preschool forms & profiles: an integrated system of multi-informant assessment. Burlington, VT: University of Vermont, Research Center for Children, Youth, and Families; 2000.
11. Adoh TO, Woodhouse JM, Oduwaiye KA. The Cardiff Test: a new visual acuity test for toddlers and children with intellectual impairment. A preliminary report. Optom Vis Sci 1992; 69(6): 427-32.
12. Fernandes M, Villar J, Stein A, et al. INTERGROWTH-21st Project international INTER-NDA standards for child development at 2 years of age: an international prospective population-based study. BMJ Open 2020; 10:e035258.
13. Kennedy S, Victora C, Craik R, et al. Deep clinical and biological phenotyping of the preterm birth and small for gestational age syndromes: The INTERBIO-21st Newborn Case-Control Study protocol [version 2; peer review: 1 approved]. Gates Open Research 2019; 2(49).
14. Kessner D, Chambers M, Burke R, Agus D, Mallick P. ProteoWizard: open source software for rapid proteomics tools development. Bioinformatics 2008; 24(21): 2534-6.
15. Watrous JD, Henglin M, Claggett B, et al. Visualization, Quantification, and Alignment of Spectral Drift in Population Scale Untargeted Metabolomics Data. Anal Chem 2017; 89(3): 1399-404.
16. Kantz ED, Tiwari S, Watrous JD, Cheng S, Jain M. Deep Neural Networks for Classification of LC-MS Spectral Peaks. Anal Chem 2019; 91(19): 12407-13.
17. McInnes L, Healy J, Melville J. UMAP: Uniform Manifold Approximation and Projection for Dimension Reduction. arXiv:180203426 2018.

Supplementary Table 1. Average posterior probabilities and odds of correct classification for fetal abdominal circumference growth trajectory group in the INTERBIO-21st Fetal Study (n=3,206).

	Faltering Growth	Median Growth	Late Accelerating	Early Accelerating
Faltering Growth	0.87	0.10	0.03	<0.01
Median Growth	0.06	0.78	0.13	0.02
Late Accelerating	0.03	0.16	0.77	0.04
Early Accelerating	<0.01	0.05	0.06	0.89
Odds of correct classification	14.3	4.9	5.9	22.3

Supplementary Table 2. Birth, neonatal outcomes, neurobehavioral outcomes and child anthropometric measures according to fetal abdominal circumference growth phenotypes in the INTERBIO-21st Fetal Study (n=3,206).

	Faltering Growth (n=763)	Median Growth Tracking (n=1166)	Late Accelerating Growth (n=773)	Early Accelerating Growth (n=504)
**Neonatal Outcomes**				
Birth outcomes, Mean ± SD				
Weight (g)	2761 ± 493	3023 ± 465	3272 ± 453	3491 ± 491
Length (cm)	47.6 ± 2.7	48.5 ± 2.5	49.1 ± 2.1	49.8 ± 2.0
Head circumference (cm)	33.0 ± 1.8	33.6 ± 1.5	34.2 ± 1.3	34.6 ± 1.3
Weight (z-score)	-0.80 ± 0.97	-0.30 ± 0.85	0.26 ± 0.85	0.72 ± 0.85
Length (z-score)	-0.54 ± 1.19	-0.18 ± 1.06	0.13 ± 0.99	0.47 ± 0.96
Head circumference (z-score)	-0.34 ± 1.18	0.04 ± 1.01	0.49 ± 0.92	0.76 ± 0.91
Weight-for-length (z-score)	-0.89 ± 1.93	-0.26 ± 1.75	0.39 ± 2.57	0.72 ± 1.43
Gestational age (weeks)	38.9 ± 2.1	38.9 ± 2.0	39.1 ± 1.9	39.0 ± 1.8
**Growth Measures**				
Measures at 1 year, Mean ± SD				
Height (z-score)	-0.93 ± 1.22	-0.38 ± 1.22	-0.23 ± 1.26	0.02 ± 1.13
Weight (z-score)	-0.64 ± 1.13	-0.03 ± 1.15	0.26 ± 1.07	0.59 ± 1.09
Head circumference (z-score)	-0.53 ± 1.16	-0.08 ± 1.16	0.26 ± 1.10	0.54 ± 1.10
Weight-for-length (z-score)	-0.26 ± 1.15	0.19 ± 1.21	0.48 ± 1.12	0.76 ± 1.12
Measures at 2 years, Mean ± SD				
Height (z-score)	-0.88 ± 1.24	-0.37 ± 1.22	-0.10 ± 1.21	0.13 ± 1.16
Weight (z-score)	-0.60 ± 1.15	-0.01 ± 1.22	0.30 ± 1.10	0.64 ± 1.15
Head circumference (z-score)	-0.65 ± 1.24	-0.17 ± 1.15	0.25 ± 1.09	0.61 ± 1.10
Weight-for-length (z-score)	-0.19 ± 1.18	0.26 ± 1.18	0.50 ± 1.10	0.79 ± 1.09
**Neurobehavioural Outcomes**				
Neurodevelopmental Assessment Mean ± SD				
Cognitive	68.2 ± 17.3	70.0 ± 16.9	70.1 ± 17.1	72.7 ± 14.9
Language	56.4 ± 20.0	60.0 ± 21.7	61.8 ± 22.3	65.5 ± 22.8
Fine motor	87.2 ± 13.9	88.7 ± 14.3	89.8 ± 14.2	90.6 ± 11.8
Gross motor	81.4 ± 15.7	81.2 ± 15.9	82.3 ± 16.5	79.4 ± 17.2

Behavioural Outcomes, Mean ± SD				
Attentional problems	4.1 ± 2.5	4.1 ± 2.3	3.8 ± 2.3	3.5 ± 2.1
Emotional reactivity	6.1 ± 1.4	6.0 ± 3.6	5.5 ± 3.4	4.7 ± 3.3
Emotional Affect, Median (IQR)				
Positive affect	90 (60-100)	80 (60-100)	90 (60-100)	90 (70-100)
Negative affect	25 (0-50)	25 (0-50)	25 (0-50)	25 (0-25)
Vision, n (%)				
Acuity >0.4 LogMAR	117 (26.7)	130 (17.7)	41 (8.9)	17 (5.0)
Contrast sensitivity >3%	82 (19.4)	86 (12.2)	33 (7.4)	12 (3.6)

Supplementary Table 3. Perinatal morbidity outcomes according to fetal abdominal circumference growth phenotypes in the INTERBIO-21st Fetal Study (n=3,206).

	Faltering Growth (n=763)	Median Growth Tracking (n=1166)	Late Accelerating Growth (n=773)	Early Accelerating Growth (n=504)
Perinatal outcomes, n (%)				
Fetal distress	67 (8.8)	89 (6.2)	48 (6.2)	34 (6.8)
Neonatal death	3 (0.4)	7 (0.6)	3 (0.4)	2 (0.4)
Caesarean section	289 (37.9)	432 (37.1)	367 (47.5)	196 (38.9)
Preterm birth	90 (11.8)	129 (11.0)	72 (9.2)	56 (11.1)
Spontaneous preterm birth	37 (4.9)	66 (5.7)	31 (4.0)	24 (4.8)
Medically-induced preterm birth	53 (7.0)	63 (5.4)	40 (5.2)	32 (6.4)
Admission to NICU	55 (7.2)	55 (4.7)	30 (3.9)	21 (4.2)
Low birth weight (<2500g)	180 (23.9)	125 (10.8)	32 (4.2)	13 (2.6)
Small for gestational age (<10th centile)	209 (27.8)	138 (11.9)	36 (3.4)	6 (1.2)

NICU = neonatal intensive care unit

Supplementary Table 4. Adjusted growth outcomes[a] at 2 years of age for all participants and stratified by duration of breastfeeding (less than 7 months vs. 7 months or longer) in the INTERBIO-21st Fetal Study (n=2,183).

Outcome	Duration of Breast feeding	Faltering Growth (n=763)	Late Accelerating Growth (n=773)	Early Accelerating Growth (n=504)
Growth at 2 years of age[b]				
Length z-score	Any	-0.38 (-0.52, -0.24)	0.17 (0.03, 0.30)	0.38 (0.22, 0.53)
	<7 mo	-0.27 (-0.55, 0.02)	0.15 (-0.10, 0.40)	0.41 (0.13, 0.69)
	≥7 mo	-0.39 (-0.55, -0.23)	0.16 (0.00, 0.32)	0.34 (0.15, 0.52)
	p_int[c]	0.55	0.62	0.69
Weight z-score	Any	-0.47 (-0.60, -0.34)	0.22 (0.09, 0.35)	0.56 (0.42, 0.71)
	<7 mo	-0.53 (-0.79, -0.26)	0.13 (-0.10, 0.36)	0.50 (0.25, 0.75)
	≥7 mo	-0.43 (-0.58, -0.27)	0.23 (0.07, 0.38)	0.54 (0.36, 0.72)
	p_int	0.44	0.38	0.75
Head circumference z-score	Any	-0.30 (-0.42, -0.17)	0.30 (0.18, 0.43)	0.58 (0.43, 0.72)
	<7 mo	-0.35 (-0.60, -0.10)	0.11 (-0.11, 0.33)	0.45 (0.21, 0.69)
	≥7 mo	-0.26 (-0.42, -0.11)	0.36 (0.20, 0.51)	0.60 (0.42, 0.77)
	p_int	0.90	0.08	0.35
Weight-for-length z-score	Any	-0.37 (-0.50, -0.23)	0.20 (0.07, 0.33)	0.50 (0.35, 0.65)
	<7 mo	-0.50 (-0.77, -0.24)	0.12 (-0.11, 0.35)	0.46 (0.21, 0.72)
	≥7 mo	-0.31 (-0.46, -0.15)	0.20 (0.04, 0.36)	0.47 (0.29, 0.66)
	p_int	0.21	0.59	0.85

[a] Models include head circumference trajectory group, maternal education (3-level) and age at birth; preterm birth and smoking in pregnancy, and child sex and age at assessment.
[b] Adjusted β and 95% CI.
[c] p_int represents the p-value for an interaction term of breastfeeding duration (<7 months vs ≥7 months) by growth trajectory.
All comparisons are made using the Median Growth Tracking Phenotype as the reference group

Supplementary Table 5. Adjusted neurodevelopmental outcomes[a] at 2 years of age for all participants and stratified by duration of breastfeeding (less than 7 months vs. 7 months or longer) in the INTERBIO-21st Fetal Study (n=2,183).

Outcome	Duration of Breast feeding	Faltering Growth (n=763)	Late Accelerating Growth (n=773)	Early Accelerating Growth (n=504)
Neurodevelopmental Assessment[b]				
Cognitive	Any	-1.34 (-3.31, 0.63)	-1.49 (-3.39, 0.41)	1.77 (-0.40, 3.94)
	<7 mo	0.10 (-3.89, 4.08)	-1.82 (-5.28, 1.65)	1.97 (-1.88, 5.82)
	≥7 mo	-1.81 (-4.10, 0.48)	-1.38 (-3.67, 0.91)	2.02 (-0.64, 4.68)
	p_int[c]	0.59	0.69	0.70
Language	Any	-2.75 (-5.25, -0.24)	-1.05 (-3.46, 1.37)	3.61 (0.86, 6.37)
	<7 mo	-2.13 (-7.53, 3.26)	-3.47 (-8.15, 1.22)	-0.26 (-5.47, 4.95)
	≥7 mo	-2.69 (-5.52, 0.14)	-0.25 (-3.08, 2.58)	5.54 (2.25, 8.83)
	p_int	0.83	0.34	0.05
Fine Motor	Any	-0.39 (-2.03, 1.24)	-0.01 (-1.59, 1.56)	0.75 (-1.05, 2.55)
	<7 mo	-0.03 (-3.39, 3.34)	-0.52 (-3.46, 2.41)	0.20 (-3.06, 3.45)
	≥7 mo	-0.33 (-2.22, 1.55)	0.14 (-1.75, 2.02)	1.20 (-0.99, 3.39)
	p_int	0.58	0.65	0.58
Gross Motor	Any	1.23 (-0.72, 3.19)	0.83 (-1.05, 2.72)	-2.42 (-4.57, -0.27)
	<7 mo	2.21 (-2.04, 6.47)	0.91 (-2.76, 4.59)	-2.77 (-6.85, 1.32)
	≥7 mo	1.11 (-1.08, 3.30)	0.95 (-1.24, 3.14)	-1.65 (-4.20, 0.89)
	p_int	0.24	0.82	0.26
Positive Affect[d]	Any	1.01 (0.89, 1.16)	0.95 (0.83, 1.08)	1.13 (0.97, 1.33)
	<7 mo	1.23 (0.93, 1.63)	0.83 (0.65, 1.05)	0.96 (0.73, 1.27)
	≥7 mo	0.98 (0.84, 1.14)	1.01 (0.86, 1.17)	1.22 (1.01, 1.49)
	p_int	0.26	0.17	0.07
Negative Affect[d]	Any	1.12 (0.99, 1.27)	1.17 (1.03, 1.32)	0.92 (0.79, 1.07)
	<7 mo	1.11 (0.87, 1.42)	1.28 (1.02, 1.61)	1.09 (0.84, 1.42)
	≥7 mo	1.12 (0.97, 1.30)	1.13 (0.98, 1.31)	0.86 (0.71, 1.04)
	p_int	0.83	0.34	0.08

Child Behaviour[b]				
Attentional Problems	Any	0.26 (-0.01, 0.54)	-0.32 (-0.58, -0.06)	-0.80 (-1.10, -0.50)
	<7 mo	0.57 (0.04, 1.11)	-0.18 (-0.65, 0.29)	-0.52 (-1.04, 0.01)
	≥7 mo	0.20 (-0.12, 0.52)	-0.40 (-0.72, -0.08)	-0.95 (-1.33, -0.58)
	p_int	0.20	0.73	0.65
Emotional Reactivity	Any	0.35 (-0.06, 0.77)	-0.58 (-0.98, -0.18)	-1.45 (-1.91, -1.00)
	<7 mo	1.41 (0.62, 2.21)	-0.14 (-0.84, 0.55)	-1.21 (-1.99, -0.44)
	≥7 mo	0.04 (-0.44, 0.53)	-0.74 (-1.22, -0.26)	-1.54 (-2.10, -0.98)
	p_int	0.00	0.30	0.82
Vision Deficits[e]				
Acuity >0.4 LogMAR	Any	1.35 (1.08, 1.70)	0.58 (0.42, 0.81)	0.35 (0.21, 0.59)
	<7 mo	1.81 (1.19, 2.75)	0.65 (0.36, 1.15)	0.12 (0.03, 0.52)
	≥7 mo	1.19 (0.90, 1.57)	0.57 (0.38, 0.86)	0.49 (0.29, 0.84)
	p_int	0.09	0.66	0.08
Contrast sensitivity >3%	Any	1.39 (1.04, 1.85)	0.64 (0.44, 0.94)	0.37 (0.20, 0.67)
	<7 mo	1.54 (0.94, 2.52)	0.62 (0.31, 1.22)	0.39 (0.14, 1.09)
	≥7 mo	1.31 (0.93, 1.86)	0.68 (0.43, 1.07)	0.37 (0.18, 0.78)
	p_int	0.30	0.60	0.87

[a] Models include head circumference trajectory group, maternal education (3-level) and age at birth; preterm birth and smoking in pregnancy, and child sex and age at assessment.

[b] Adjusted β and 95% CI

[c] p_int represents the p-value for an interaction term of breastfeeding duration (<7 months vs ≥7 months) by growth trajectory.

[d] Adjusted Incidence Rate Ratio and 95% CI

[e] Adjusted Relative Risk and 95% CI

All comparisons are made using the Median Growth Tracking Phenotype as the reference group

Supplementary Table 6. Maternal characteristics according to fetal abdominal circumference trajectory phenotypes in the INTERBIO-21st Fetal Study (n=3,206).

Condition	Faltering Growth (n=763)	Median Growth Tracking (n=1166)	Late Accelerating Growth (n=773)	Early Accelerating Growth (n=504)
Age (years)	28.3 ± 5.9	28.9 ± 5.4	29.8 ± 5.0	30.4 ± 4.8
Married or cohabitating	606 (79.4)	1010 (86.6)	670 (86.7)	458 (90.9)
University education	221 (29.0)	443 (38.0)	340 (44.0)	239 (47.4)
Smoking during pregnancy	46 (6.0)	79 (6.8)	22 (2.9)	15 (3.0)
Alcohol use during pregnancy	19 (2.5)	24 (2.1)	19 (2.5)	14 (2.8)
Previous parity	479 (60.7)	671 (57.6)	469 (60.7)	286 (56.8)
Maternal height (cm)	156.9 ± 6.9	159.5 ± 7.3	160.7 ± 7.0	164.1 ± 7.2
Pre-pregnancy weight (kg)	59.0 ± 13.0	61.7 ± 12.6	66.6 ± 12.2	69.0 ± 12.5
BMI (kg/m^2)	23.8 ± 4.4	24.2 ± 4.2	25.8 ± 4.2	25.6 ± 4.3
Obese (BMI≥30)	82 (10.8)	131 (11.2)	138 (17.9)	91 (18.1)
Overweight or obese (BMI≥25)	287 (37.6)	459 (39.4)	425 (55.0)	259 (51.4)
History of diabetes	13 (1.7)	18 (1.5)	23 (3.0)	9 (1.8)
History of hypertension	27 (3.5)	37 (3.2)	33 (4.3)	18 (3.6)
History of thyroid/endocrine condition	38 (5.0)	86 (7.4)	75 (9.7)	51 (10.1)
Diabetes/endocrine diagnosed/treated during pregnancy	35 (4.6)	70 (6.0)	59 (7.6)	39 (7.7)
Gestational diabetes	37 (4.9)	73 (6.3)	51 (6.6)	36 (7.1)
Pregnancy-induced hypertension	48 (6.3)	56 (4.8)	62 (8.0)	49 (9.7)
Preeclampsia/eclampsia/HELLP	25 (3.3)	33 (2.8)	17 (2.2)	11 (2.2)

BMI = body mass index; HELLP = Haemolysis, Elevated Liver enzymes and Low Platelets

Supplementary Table 7. Unadjusted growth outcomes at 2 years of age stratified by duration of breastfeeding (less than 7 months vs. 7 months or longer) in the INTERBIO-21st Fetal Study (N=2,183).

Outcome	Duration of Breast feeding	Faltering Growth (n=763)	Late Accelerating Growth (n=773)	Early Accelerating Growth (n=504)
Growth at 2 years of age[a]				
Length z-score	Any	-0.51 (-0.65, -0.37)	0.27 (0.13, 0.40)	0.50 (0.35, 0.65)
	<7 mo	-0.46 (-0.73, -0.18)	0.16 (-0.08, 0.41)	0.49 (0.23, 0.76)
	≥7 mo	-0.51 (-0.67, -0.35)	0.29 (0.13, 0.46)	0.48 (0.30, 0.67)
	p_int[b]	0.73	0.39	0.95
Weight z-score	Any	-0.59 (-0.72, -0.46)	0.32 (0.19, 0.44)	0.66 (0.51, 0.80)
	<7 mo	-0.68 (-0.93, -0.43)	0.18 (-0.04, 0.41)	0.58 (0.34, 0.82)
	≥7 mo	-0.55 (-0.70, -0.39)	0.36 (0.20, 0.51)	0.66 (0.49, 0.84)
	p_int	0.41	0.22	0.61
Head circumference z-score	Any	-0.47 (-0.60, -0.34)	0.43 (0.30, 0.56)	0.78 (0.64, 0.92)
	<7 mo	-0.45 (-0.69, -0.20)	0.24 (0.02, 0.45)	0.65 (0.41, 0.88)
	≥7 mo	-0.47 (-0.63, -0.32)	0.50 (0.35, 0.66)	0.83 (0.66, 1.01)
	p_int	0.87	0.06	0.23
Weight-for-length z-score	Any	-0.44 (-0.57, -0.31)	0.25 (0.12, 0.37)	0.53 (0.39, 0.68)
	<7 mo	-0.58 (-0.83, -0.33)	0.16 (-0.07, 0.38)	0.49 (0.25, 0.74)
	≥7 mo	-0.39 (-0.55, -0.24)	0.27 (0.12, 0.43)	0.53 (0.36, 0.71)
	p_int	0.23	0.42	0.79

[a] Unadjusted β and 95% CI.
[b] p_int represents the p-value for an interaction term of breastfeeding duration (<7 months vs ≥7 months) by growth trajectory.
All comparisons are made using the Median Growth Tracking Phenotype as the reference group

Supplementary Table 8. Unadjusted neurodevelopmental outcomes at 2 years of age for all participants and stratified by duration of breastfeeding (less than 7 months vs. 7 months or longer) in the INTERBIO-21st Fetal Study (n=2,183).

Outcome	Duration of Breast Feeding	Faltering Growth (n=763)	Late Accelerating Growth (n=773)	Early Accelerating Growth (n=504)
Neurodevelopmental Assessment[a]				
Cognitive	Any	-1.81 (-3.74, 0.13)	0.03 (-1.87, 1.94)	2.72 (0.61, 4.84)
	<7 mo	-1.31 (-5.15, 2.52)	-0.52 (-3.95, 2.91)	2.41 (-1.30, 6.12)
	≥7 mo	-1.98 (-4.24, 0.28)	0.31 (-1.99, 2.60)	2.92 (0.33, 5.50)
	p_int[b]	0.77	0.70	0.83
Language	Any	-3.69 (-6.21, -1.17)	1.78 (-0.69, 4.25)	5.43 (2.68, 8.17)
	<7 mo	-3.57 (-8.88, 1.74)	-0.25 (-5.00, 4.50)	1.81 (-3.33, 6.96)
	≥7 mo	-3.60 (-6.45, -0.76)	2.54 (-0.36, 5.44)	7.07 (3.81, 10.33)
	p_int	0.99	0.31	0.08
Fine Motor	Any	-1.48 (-3.09, 0.12)	1.07 (-0.50, 2.65)	1.83 (0.08, 3.58)
	<7 mo	-2.33 (-5.58, 0.92)	0.18 (-2.74, 3.10)	0.63 (-2.51, 3.78)
	≥7 mo	-1.15 (-3.01, 0.70)	1.41 (-0.47, 3.30)	2.38 (0.27, 4.50)
	p_int	0.54	0.48	0.26
Gross Motor	Any	0.22 (-1.66, 2.11)	1.14 (-0.71, 2.99)	-1.72 (-3.77, 0.34)
	<7 mo	2.57 (-1.42, 6.57)	0.79 (-2.78, 4.35)	-3.61 (-7.47, 0.25)
	≥7 mo	-0.44 (-2.56, 1.69)	1.39 (-0.78, 3.55)	-0.65 (-3.08, 1.79)
	p_int	0.18	0.77	0.19
Positive Affect[c]	Any	1.02 (0.90, 1.16)	1.01 (0.88, 1.14)	1.18 (1.02, 1.38)
	<7 mo	1.18 (0.89, 1.56)	0.89 (0.70, 1.13)	0.99 (0.76, 1.29)
	≥7 mo	0.99 (0.86, 1.14)	1.06 (0.91, 1.24)	1.29 (1.07, 1.55)
	p_int	0.28	0.21	0.12
Negative Affect[c]	Any	1.11 (0.99, 1.25)	1.08 (0.96, 1.22)	0.87 (0.75, 1.01)
	<7 mo	1.09 (0.85, 1.38)	1.17 (0.94, 1.47)	1.03 (0.80, 1.33)
	≥7 mo	1.11 (0.97, 1.27)	1.04 (0.90, 1.20)	0.81 (0.67, 0.97)
	p_int	0.89	0.37	0.13

Child Behaviour[a]				
Attentional Problems	Any	0.00 (-0.27, 0.27)	-0.32 (-0.58, -0.05)	-0.61 (-0.90, -0.32)
	<7 mo	0.30 (-0.22, 0.81)	-0.36 (-0.82, 0.10)	-0.72 (-1.23, -0.22)
	≥7 mo	-0.09 (-0.40, 0.23)	-0.32 (-0.64, -0.01)	-0.58 (-0.94, -0.22)
	p_int	0.22	0.91	0.65
Emotional Reactivity	Any	0.12 (-0.28, 0.53)	-0.51 (-0.91, -0.11)	-1.29 (-1.73, -0.85)
	<7 mo	1.42 (0.65, 2.18)	-0.40 (-1.09, 0.28)	-1.70 (-2.45, -0.96)
	≥7 mo	-0.31 (-0.79, 0.16)	-0.57 (-1.06, -0.09)	-1.06 (-1.60, -0.51)
	p_int	0.00	0.69	0.18
Vision Deficits[d]				
Acuity >0.4 LogMAR	Any	1.50 (1.21, 1.87)	0.50 (0.36, 0.70)	0.28 (0.17, 0.46)
	<7 mo	2.27 (1.54, 3.35)	0.58 (0.33, 1.03)	0.10 (0.02, 0.41)
	≥7 mo	1.27 (0.97, 1.67)	0.47 (0.31, 0.71)	0.37 (0.22, 0.63)
	p_int	0.02	0.54	0.09
Contrast sensitivity >3%	Any	1.59 (1.20, 2.10)	0.61 (0.42, 0.89)	0.30 (0.17, 0.54)
	<7 mo	2.24 (1.39, 3.59)	0.53 (0.26, 1.06)	0.28 (0.10, 0.77)
	≥7 mo	1.37 (0.97, 1.93)	0.65 (0.41, 1.03)	0.31 (0.15, 0.63)
	p_int	0.10	0.63	0.87

[a] Unajusted β and 95% CI

[b] p_int represents the p-value for an interaction term of breastfeeding duration (<7 months vs ≥7 months) by growth trajectory.

[c] Unadjusted Incidence Rate Ratio and 95% CI

[d] Unadjusted Relative Risk and 95% CI

All comparisons are made using the Median Growth Tracking Phenotype as the reference group

Supplementary Fig. 1. INTERBIO-21st Fetal Study participant flow chart from early pregnancy to 2 years of age.

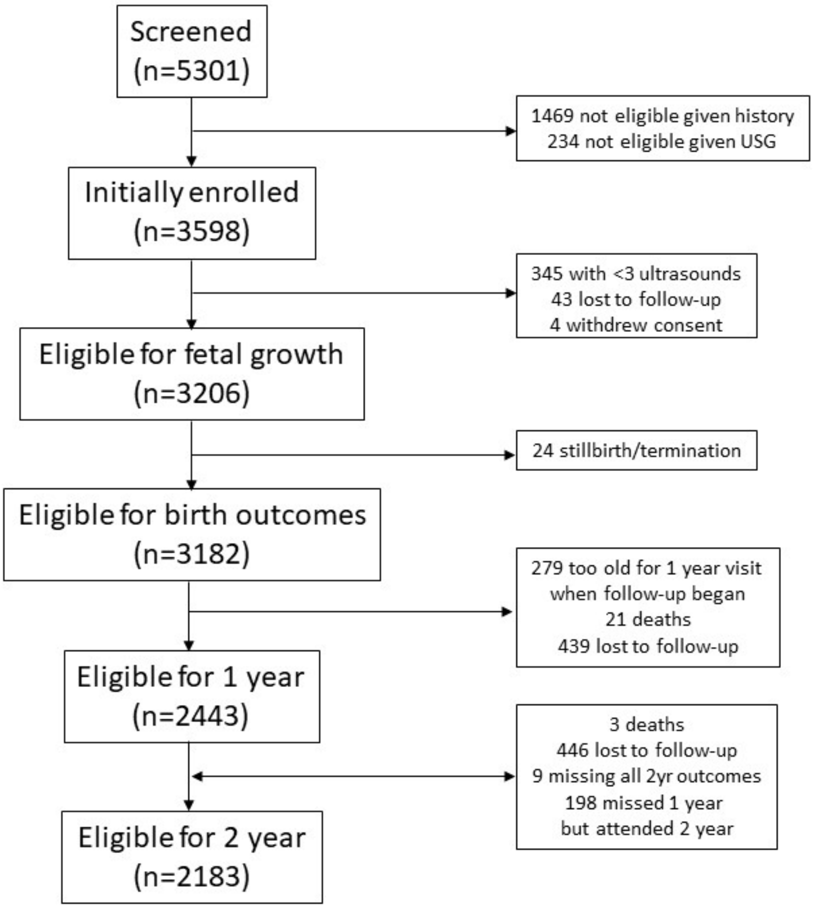

Supplementary Fig. 2. INTERBIO-21st Fetal Study directed acyclic graph for abdominal growth trajectory group and neurodevelopment at 2 years of age.

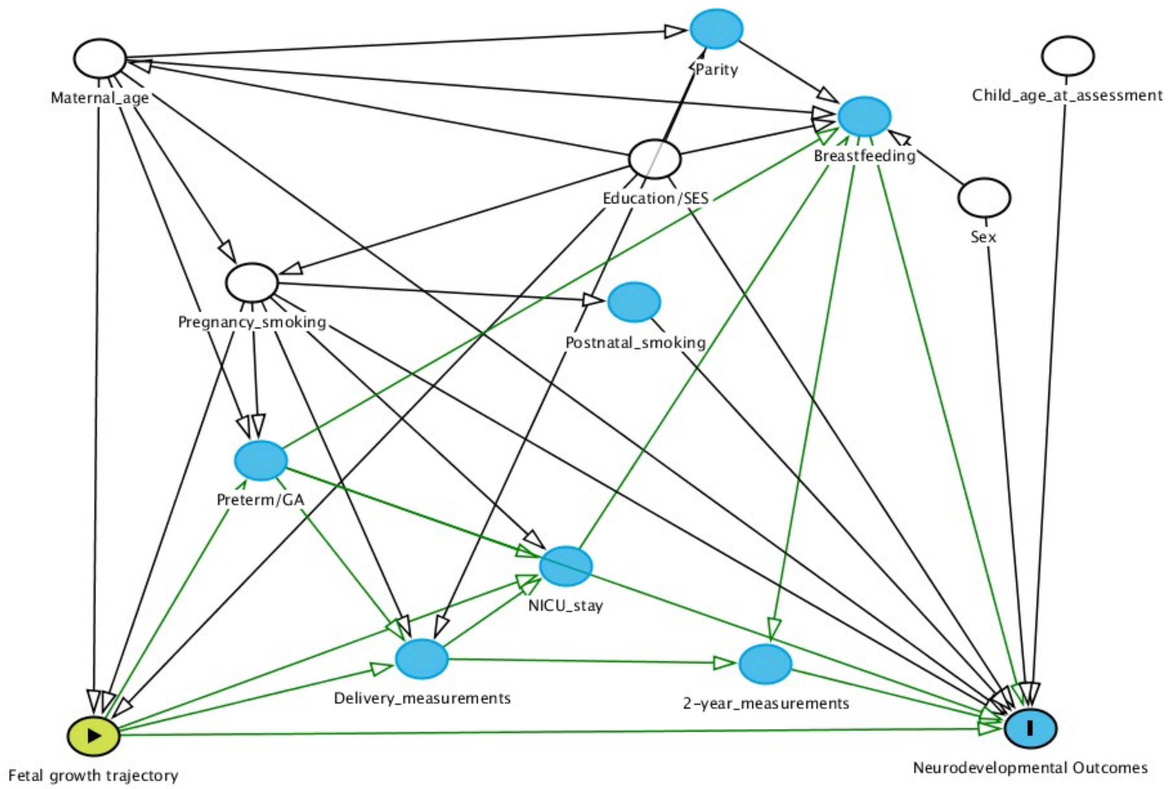

Supplementary Fig. 3. Changes in growth outcomes at 2 years of age associated with fetal abdominal circumference growth phenotypes, stratified by duration of breastfeeding in the INTERBIO-21st Fetal Study (n=2,183).

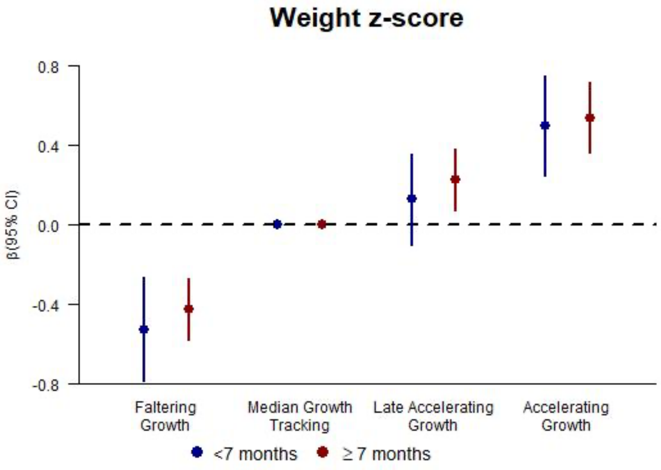

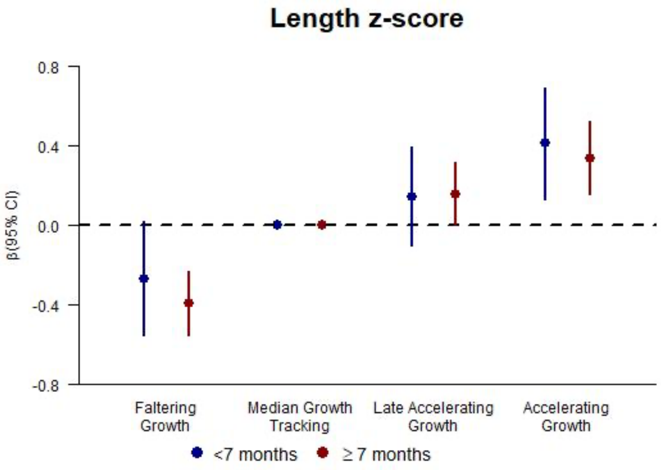

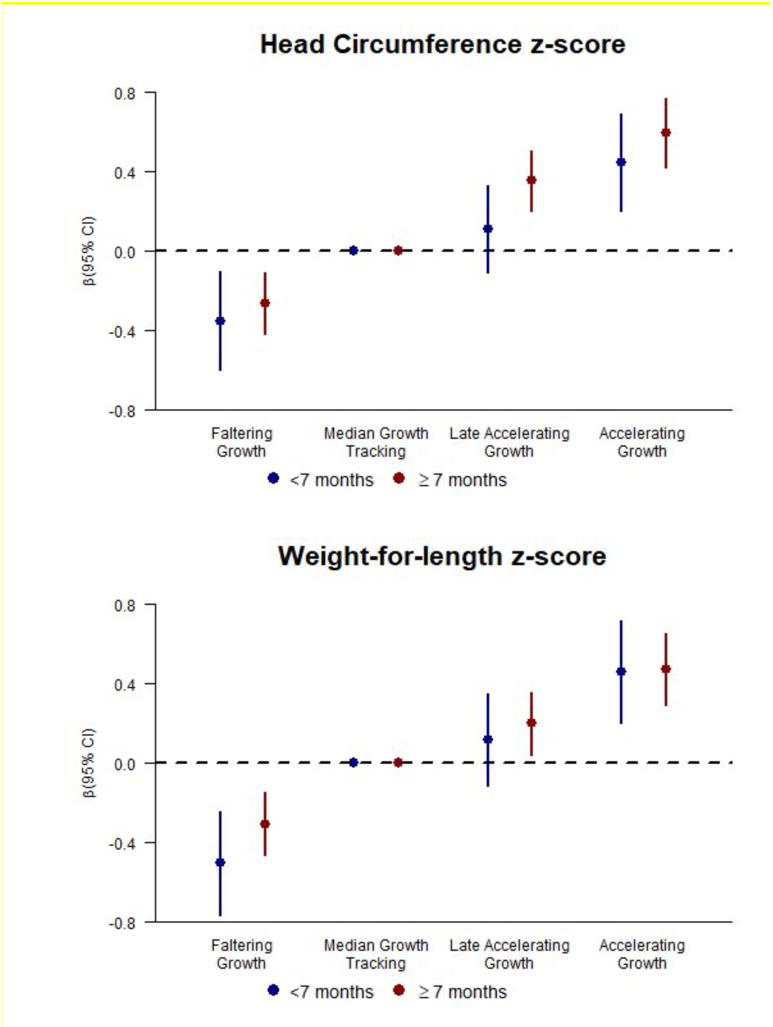
Head Circumference z-score
0.8
0.4
0.0
-0.4
-0.8
β(95% CI)
Faltering Growth
Median Growth Tracking
Late Accelerating Growth
Accelerating Growth
<7 months
≥ 7 months
Weight-for-length z-score
0.8
0.4
0.0
-0.4
-0.8
β(95% CI)
Faltering Growth
Median Growth Tracking
Late Accelerating Growth
Accelerating Growth
<7 months
≥ 7 months

Supplementary Fig. 4. Supervised Uniform Manifold Approximation and Projection for the Faltering Growth (red dots) and Early Accelerating Growth (blue dots) phenotypes of ultrasound-based, fetal abdominal circumference growth trajectories, (n=2,713 women; 2,430 newborns).

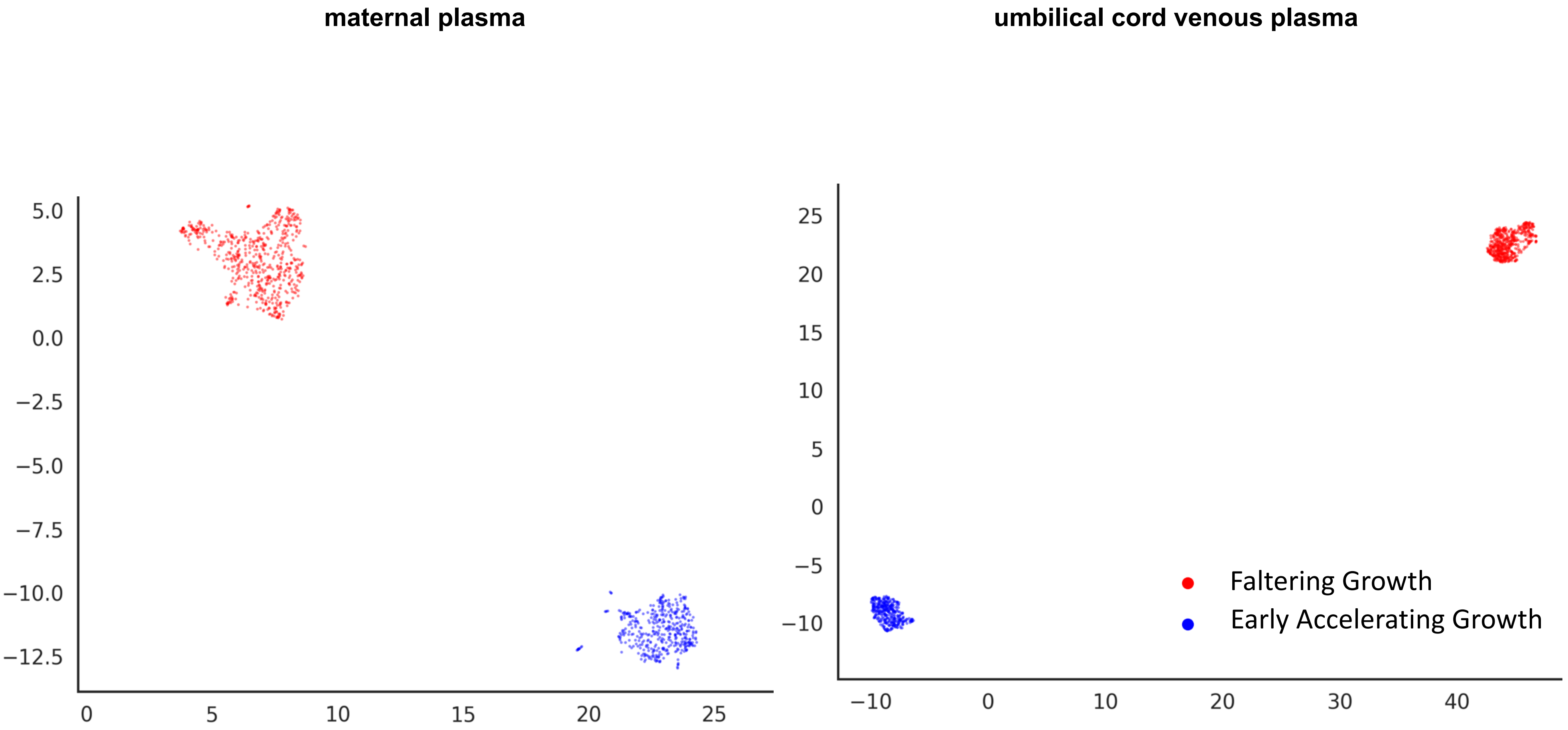

Supplementary Fig. 5. Growth trajectories for Faltering Growth and Early Accelerating Growth phenotypes, stratified by study site, in the INTERBIO-21st Fetal Study (n=3,206).

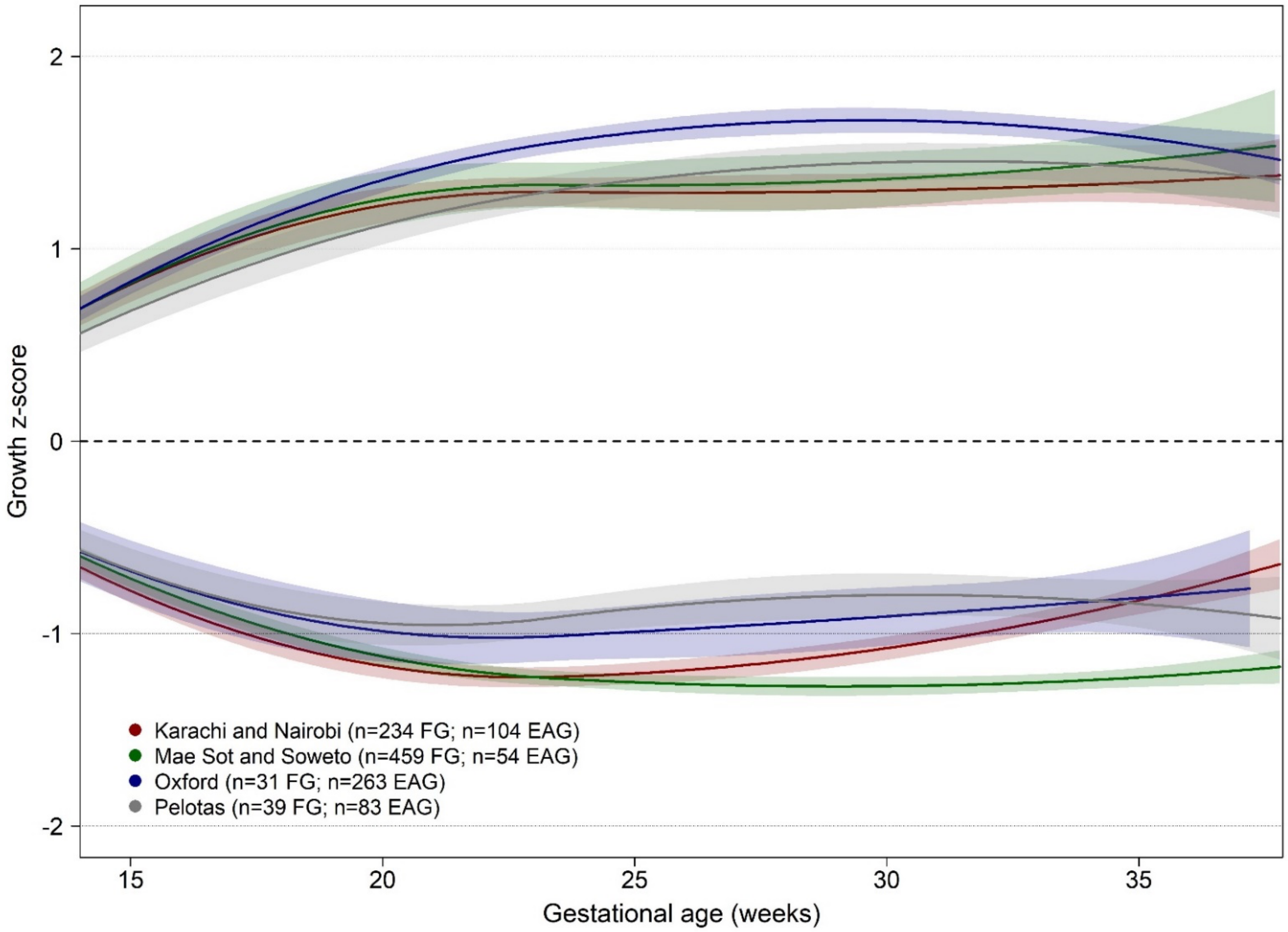

Shaded bands represent 95% confidence intervals for splines used to summarise group trajectories.

Supplementary Fig. 6. INTERBIO-21st Fetal Study metabolic features flow chart.

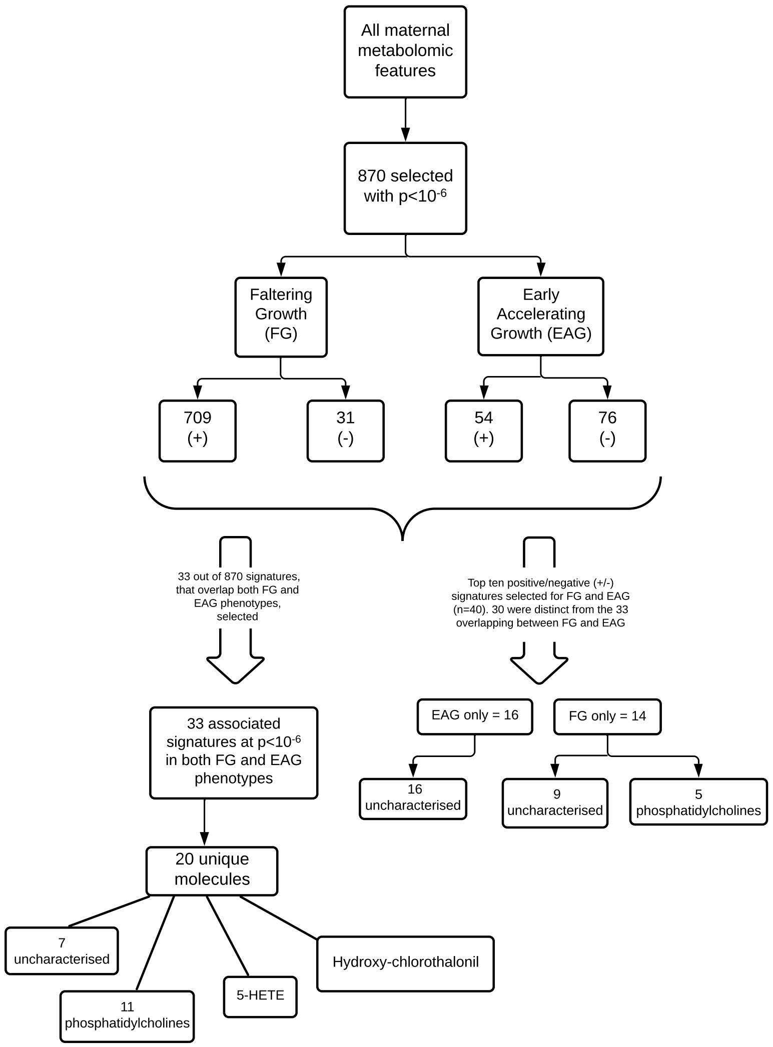

Supplementary Fig. 7. Fetal abdominal and head circumference accelerating and faltering growth phenotypes in the INTERBIO-21st Fetal Study (n=3,206).

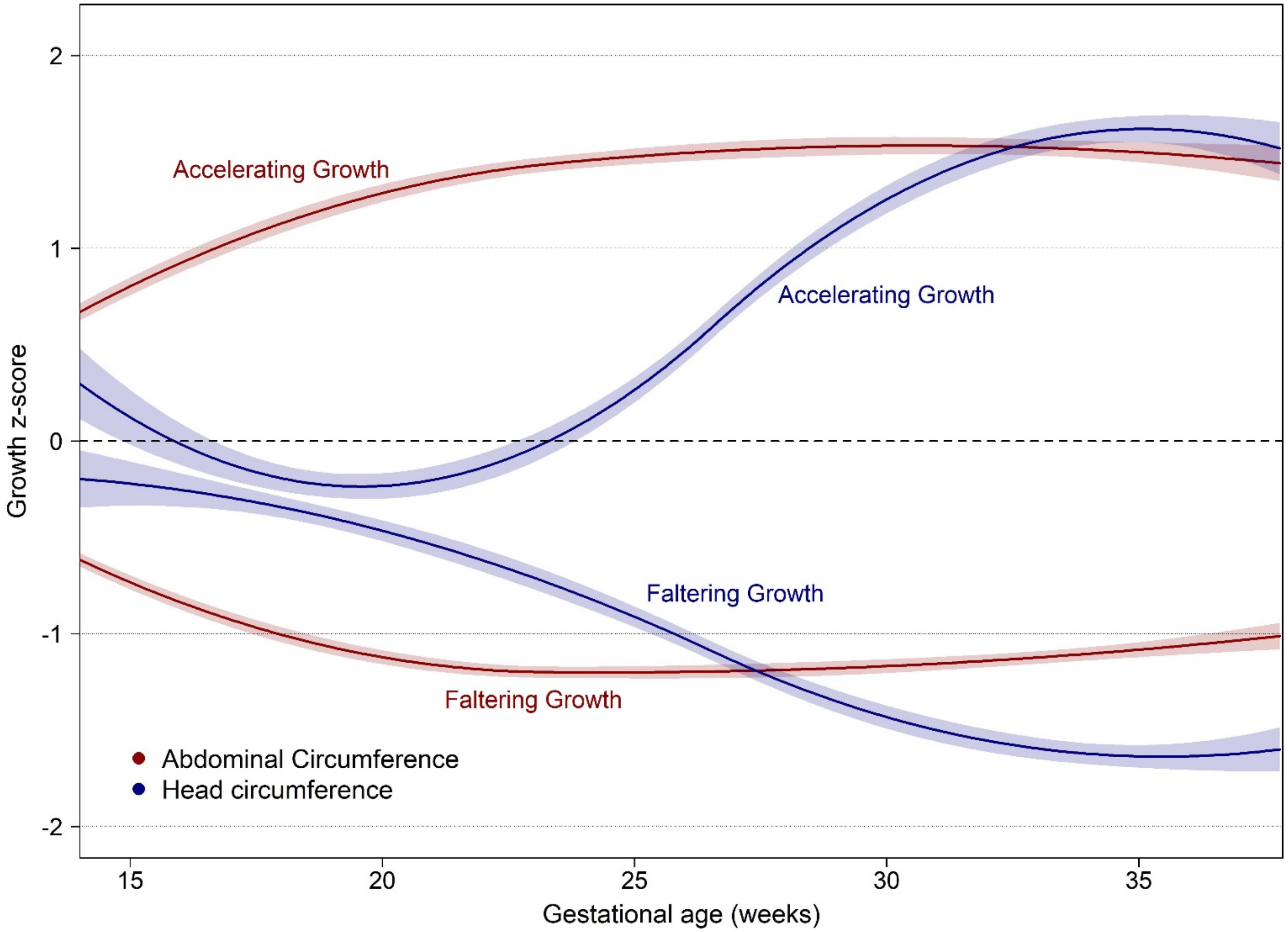

Shaded bands represent 95% confidence intervals for splines used to summarise group trajectories.

Metabolomic data

Metabolite feature label	MZ
709 metabolites	
pos_mtb_3701021	832.6331
pos_mtb_3701041	833.6365
pos_mtb_3784852	831.634
neg_mtb_2712773	388.3276
pos_mtb_470809	188.0554
pos_mtb_2220387	254.1174
pos_mtb_546689	142.0492
pos_mtb_2498669	790.5615
pos_mtb_3025995	844.5455
pos_mtb_3559591	789.5608
pos_mtb_2913737	756.5535
pos_mtb_2394295	806.5663
neg_mtb_2663874	900.595
pos_mtb_2289614	830.5549
pos_mtb_1947041	964.4481
pos_mtb_2316054	878.5513
pos_mtb_2989645	796.5474
neg_mtb_2722709	882.5838
pos_mtb_2809146	540.3141
pos_mtb_2614468	774.5635
pos_mtb_2367731	792.5916
pos_mtb_2945135	780.5517
pos_mtb_2970668	757.5566
pos_mtb_3273976	848.5756
pos_mtb_3062445	798.5637
pos_mtb_2616233	902.5135
pos_mtb_2703356	880.5143
pos_mtb_2915004	836.5563
pos_mtb_3367644	826.5922
pos_mtb_2728255	903.5145
pos_mtb_2528916	782.5671
neg_mtb_2767708	857.573
pos_mtb_2840664	830.5663
neg_mtb_2568345	317.2477
pos_mtb_2117238	845.5362
neg_mtb_2687767	834.5732
pos_mtb_2614483	775.5671
pos_mtb_470827	205.0816
pos_mtb_2840040	773.5519
pos_mtb_2990081	822.5621
pos_mtb_2498520	780.5535
pos_mtb_547547	309.1291
pos_mtb_2786636	862.5551
pos_mtb_2557521	804.5533
pos_mtb_2702238	785.587

pos_mtb_3009920	820.5463
neg_mtb_2708600	387.326
neg_mtb_2391504	359.2189
neg_mtb_2877020	867.6524
pos_mtb_2136999	874.5663
pos_mtb_2267015	784.5781
pos_mtb_2528908	781.5565
pos_mtb_3110009	876.6057
neg_mtb_2718815	880.5712
pos_mtb_2499146	819.5332
pos_mtb_2756600	772.5482
neg_mtb_2673765	872.557
pos_mtb_3274003	851.5955
pos_mtb_2971161	799.5671
pos_mtb_1946759	943.4675
neg_mtb_2172114	417.2257
neg_mtb_2761177	856.5713
pos_mtb_2442584	818.5289
pos_mtb_3559219	764.5573
pos_mtb_2009522	840.5664
pos_mtb_3579162	765.561
pos_mtb_3367623	824.5773
pos_mtb_2971442	823.5659
pos_mtb_2212281	942.4724
neg_mtb_2673466	832.5712
pos_mtb_3376556	849.5782
neg_mtb_2688809	990.5559
neg_mtb_2429222	335.2224
pos_mtb_3133757	825.5821
pos_mtb_3043624	875.5944
neg_mtb_2767902	909.6031
neg_mtb_2588704	901.5269
pos_mtb_3179233	872.575
pos_mtb_3042675	800.5777
pos_mtb_2315497	850.5861
pos_mtb_2043123	860.5419
pos_mtb_2443229	864.5718
pos_mtb_3178967	847.5563
pos_mtb_3200711	846.5569
neg_mtb_2749296	767.5378
neg_mtb_2334455	679.2133
pos_mtb_2588556	797.5494
neg_mtb_2748699	667.4715
neg_mtb_2773632	669.4868
neg_mtb_2786143	693.5268
neg_mtb_2678182	833.5737
neg_mtb_2687993	860.5933
pos_mtb_2589381	855.5083
neg_mtb_2677740	766.5335

pos_mtb_2727687	864.5685
pos_mtb_3042910	821.5486
neg_mtb_2682193	764.5333
pos_mtb_3179244	873.5771
neg_mtb_2702110	884.5844
pos_mtb_2945408	797.5514
pos_mtb_3158137	888.6363
pos_mtb_2915171	845.5513
neg_mtb_2436870	401.231
neg_mtb_2741057	908.5983
pos_mtb_2728287	905.5244
pos_mtb_2588781	812.5395
pos_mtb_2915302	852.5976
pos_mtb_2616761	938.4754
pos_mtb_2042860	838.5443
pos_mtb_3041147	657.5058
pos_mtb_2289352	814.5576
neg_mtb_2379782	419.2438
pos_mtb_522362	255.0651
pos_mtb_2671615	854.5246
neg_mtb_2742762	197.8073
neg_mtb_2761466	910.6077
pos_mtb_2468405	912.4561
pos_mtb_3604985	790.5728
pos_mtb_2840352	802.5937
pos_mtb_2971259	808.5769
pos_mtb_2290271	876.5352
pos_mtb_2787690	940.494
pos_mtb_2064221	904.5649
pos_mtb_1765828	258.1259
pos_mtb_2442791	832.5829
neg_mtb_2449929	317.212
pos_mtb_2529240	805.5561
pos_mtb_2873165	865.5741
neg_mtb_2767571	810.5623
neg_mtb_2682875	858.5917
pos_mtb_2616581	926.5469
neg_mtb_2769953	548.4059
neg_mtb_2572625	874.5754
pos_mtb_2758532	894.5031
pos_mtb_2529974	858.5845
pos_mtb_3670601	914.644
pos_mtb_2814559	881.5459
pos_mtb_2344942	853.5422
pos_mtb_3320751	892.5814
pos_mtb_2558954	875.5848
pos_mtb_2590215	914.4632
neg_mtb_2473930	301.2167
pos_mtb_587122	132.0654

neg_mtb_2125768	435.2384
neg_mtb_2673578	848.5446
neg_mtb_2740877	876.5751
pos_mtb_2009177	800.5842
pos_mtb_2023515	837.5546
neg_mtb_2623354	898.5771
pos_mtb_2289910	850.5169
pos_mtb_2786490	852.5543
neg_mtb_2522396	344.2318
neg_mtb_2617870	888.5615
pos_mtb_3062891	831.5552
neg_mtb_2687292	778.5027
neg_mtb_2450482	402.2336
neg_mtb_2687148	758.5325
pos_mtb_3109431	839.5748
pos_mtb_2290197	870.5471
neg_mtb_2617783	873.5649
pos_mtb_367735	226.0826
pos_mtb_2178916	866.5839
neg_mtb_2449954	320.2313
pos_mtb_6193131	925.7139
pos_mtb_367613	208.0819
pos_mtb_479325	202.0824
pos_mtb_2614994	813.5449
neg_mtb_2722704	881.5732
pos_mtb_2590247	916.5046
pos_mtb_2089616	839.5573
pos_mtb_2669770	762.4939
pos_mtb_2117800	902.5952
neg_mtb_2186780	418.2285
neg_mtb_2201571	332.0392
pos_mtb_2786059	819.593
pos_mtb_2239046	375.2147
pos_mtb_2757727	845.5486
pos_mtb_1967901	942.4663
neg_mtb_2429226	336.2257
pos_mtb_2528929	783.5719
pos_mtb_2726286	765.5618
neg_mtb_2596004	900.5868
neg_mtb_2745810	924.5839
pos_mtb_2227768	956.4951
pos_mtb_2671785	863.5578
pos_mtb_2970834	770.5856
pos_mtb_2971181	801.5773
pos_mtb_2786241	833.5865
neg_mtb_2742856	221.8517
neg_mtb_2302162	663.2265
pos_mtb_2004192	181.0858
neg_mtb_2756952	691.5128

neg_mtb_2458019	319.2278
pos_mtb_3110110	883.5431
pos_mtb_3157403	822.562
pos_mtb_2913914	766.5562
neg_mtb_2714511	788.5234
neg_mtb_2697728	873.5366
pos_mtb_2466570	809.5869
neg_mtb_2786559	766.5545
pos_mtb_2315320	841.5754
neg_mtb_2771031	887.617
pos_mtb_5538792	909.7147
neg_mtb_2688242	896.5649
pos_mtb_2670044	776.5725
pos_mtb_2558292	843.5932
neg_mtb_2705878	759.5567
pos_mtb_2873470	879.5645
pos_mtb_5569214	775.5359
pos_mtb_2589468	861.5443
neg_mtb_2761341	886.6133
pos_mtb_3350384	870.598
neg_mtb_2753737	806.5558
pos_mtb_2640875	860.5389
neg_mtb_2783133	909.5857
pos_mtb_2465700	754.537
neg_mtb_2722983	936.6041
pos_mtb_432230	235.0927
pos_mtb_2871745	795.5438
pos_mtb_3009914	819.5341
neg_mtb_2767882	901.5973
neg_mtb_2778170	811.5874
neg_mtb_2745220	820.5249
pos_mtb_3757971	913.6353
neg_mtb_2781226	462.3664
pos_mtb_3605014	791.5761
neg_mtb_2639411	913.5663
pos_mtb_2912410	655.4911
pos_mtb_2498684	791.5631
pos_mtb_2394736	871.5529
pos_mtb_3638145	891.7065
pos_mtb_2756930	793.5922
neg_mtb_2742716	179.8413
neg_mtb_2465996	321.2425
neg_mtb_2753745	807.5425
pos_mtb_3025373	794.6036
pos_mtb_2558565	856.5052
neg_mtb_2644898	914.5742
pos_mtb_3514737	716.5572
neg_mtb_2332116	229.0338
pos_mtb_3009453	781.5549

neg_mtb_2431210	641.2155
neg_mtb_2715291	964.585
neg_mtb_2415921	723.2161
pos_mtb_3044643	969.5159
pos_mtb_2971836	853.6081
neg_mtb_2682531	808.5273
neg_mtb_2726978	883.5872
neg_mtb_1953032	411.2382
pos_mtb_2150728	301.2159
pos_mtb_3236413	655.4924
neg_mtb_2580770	889.5561
pos_mtb_1946061	887.5458
pos_mtb_2673310	936.4637
neg_mtb_2650557	874.5845
pos_mtb_453722	172.0621
pos_mtb_2211489	859.5548
neg_mtb_2701961	859.5876
neg_mtb_2110347	619.2069
neg_mtb_2747014	201.8019
neg_mtb_2259411	436.2432
pos_mtb_2615636	859.5867
neg_mtb_1011734	277.0678
pos_mtb_2290885	922.5463
pos_mtb_6573291	941.6969
pos_mtb_3350493	875.5862
pos_mtb_3129908	485.3611
pos_mtb_2756523	767.5759
pos_mtb_2225695	778.5376
neg_mtb_2529201	343.2279
neg_mtb_2588611	890.5676
neg_mtb_2145203	827.2371
pos_mtb_2394589	847.6272
neg_mtb_2443388	426.2352
neg_mtb_2742758	195.8108
pos_mtb_2369810	894.546
pos_mtb_3405678	766.5728
neg_mtb_2701374	763.5121
neg_mtb_2742768	199.8043
pos_mtb_3396496	784.4884
pos_mtb_2179243	892.5325
neg_mtb_2414568	565.2137
neg_mtb_2701589	804.5226
pos_mtb_2911985	631.4916
neg_mtb_2437304	477.2175
pos_mtb_2991795	936.5165
pos_mtb_3350826	893.5851
pos_mtb_3157202	801.5829
pos_mtb_387237	255.0647
pos_mtb_3597155	445.3714

pos_mtb_2237972	245.1862
neg_mtb_2693732	982.5946
neg_mtb_2171175	315.1961
neg_mtb_2707076	962.5741
neg_mtb_2216480	373.1985
pos_mtb_3368254	892.577
neg_mtb_1999790	597.2242
neg_mtb_2202961	493.3018
neg_mtb_2158913	605.2333
neg_mtb_2596645	978.5652
neg_mtb_1583598	-
pos_mtb_2179042	877.5374
pos_mtb_3063927	906.5366
neg_mtb_2414525	559.2239
pos_mtb_2616463	918.5031
neg_mtb_2783047	885.5955
pos_mtb_1940148	342.156
neg_mtb_2636664	528.1953
pos_mtb_307093	264.1125
neg_mtb_2607789	899.5771
pos_mtb_1962424	364.1373
neg_mtb_2828847	447.3775
pos_mtb_3156939	773.5528
pos_mtb_1988382	965.4468
neg_mtb_2748712	668.4734
neg_mtb_2595209	812.5144
pos_mtb_1908363	343.1616
neg_mtb_801725	277.0752
neg_mtb_2626102	520.1413
pos_mtb_4390439	907.7043
neg_mtb_2742726	181.8378
pos_mtb_6609669	952.7261
neg_mtb_2749522	826.5541
pos_mtb_2559816	917.4963
pos_mtb_2499435	834.5253
neg_mtb_2705928	762.5073
neg_mtb_2274302	441.2247
neg_mtb_2773638	672.4848
pos_mtb_2499450	835.5281
pos_mtb_2700703	631.4878
pos_mtb_2195779	779.5413
neg_mtb_2596465	956.5773
neg_mtb_2735642	866.5856
pos_mtb_2617293	974.5668
pos_mtb_3545449	767.5786
pos_mtb_3350406	871.6042
neg_mtb_2158715	582.2328
neg_mtb_2638976	854.5464
neg_mtb_2658123	1007.635

pos_mtb_2787982	962.4843
pos_mtb_3560536	855.6335
pos_mtb_3373880	470.3681
pos_mtb_2557740	816.5731
neg_mtb_2644795	891.6163
pos_mtb_3085210	830.6144
neg_mtb_2715100	914.5847
pos_mtb_3110019	877.6074
pos_mtb_5701661	910.7172
pos_mtb_2561001	975.5656
neg_mtb_2736640	1037.656
pos_mtb_3009387	774.5636
pos_mtb_2112668	431.2043
pos_mtb_3156923	772.5476
neg_mtb_2726268	739.5123
pos_mtb_2639561	771.5661
pos_mtb_2408455	671.4863
pos_mtb_1885437	853.5569
pos_mtb_2972803	942.4955
pos_mtb_2192372	391.2112
pos_mtb_2557967	828.543
neg_mtb_2731577	966.6147
neg_mtb_2655985	731.5146
pos_mtb_2988064	671.4758
neg_mtb_2744493	683.4654
pos_mtb_4074500	766.5734
pos_mtb_3559792	805.5552
neg_mtb_2668271	794.5344
neg_mtb_2442804	318.2153
neg_mtb_2688274	905.5858
neg_mtb_2767735	861.5979
pos_mtb_2758945	920.5656
pos_mtb_2702048	768.5748
pos_mtb_3108873	799.5679
pos_mtb_2640404	829.5631
pos_mtb_2641985	937.4741
pos_mtb_2137572	924.5466
pos_mtb_3558882	743.5783
pos_mtb_7088273	951.7242
neg_mtb_1202037	325.1743
pos_mtb_2196034	803.5423
pos_mtb_2990099	823.5553
pos_mtb_2971867	856.5056
pos_mtb_587507	276.155
pos_mtb_1961191	186.1488
pos_mtb_2441871	755.5333
neg_mtb_2745519	877.5853
neg_mtb_2825654	295.2273
pos_mtb_1879172	383.2185

neg_mtb_2668443	823.5362
neg_mtb_2865161	868.6555
neg_mtb_2612316	844.5342
pos_mtb_3008459	673.5027
pos_mtb_2245030	829.5432
pos_mtb_2179860	943.4726
neg_mtb_2757047	714.4847
neg_mtb_2288001	581.2267
pos_mtb_547642	318.1287
pos_mtb_6329214	933.7129
neg_mtb_2285381	243.1716
neg_mtb_2761434	902.6055
pos_mtb_2409849	758.567
pos_mtb_3558867	742.5751
pos_mtb_3756804	809.6481
pos_mtb_2560474	950.5668
neg_mtb_2128767	745.236
pos_mtb_2758158	871.6248
neg_mtb_2678245	842.5541
neg_mtb_2322039	351.218
pos_mtb_1987253	886.5446
neg_mtb_2711276	939.5761
pos_mtb_2529394	818.5853
pos_mtb_2238727	343.1578
pos_mtb_2810497	629.4751
pos_mtb_2911730	616.5015
pos_mtb_2642022	940.4751
pos_mtb_2369020	855.5859
pos_mtb_2064233	905.5741
neg_mtb_2673112	779.5248
neg_mtb_993416	605.076
neg_mtb_2595674	864.5135
neg_mtb_2731066	880.5851
neg_mtb_2698256	982.6033
pos_mtb_3105413	486.3637
pos_mtb_2171684	263.0886
neg_mtb_2692682	809.5341
neg_mtb_2735543	852.5558
neg_mtb_2682626	815.5554
pos_mtb_2615893	876.5139
neg_mtb_2722311	794.5457
pos_mtb_3373799	431.3783
neg_mtb_1908775	583.118
neg_mtb_2745141	794.5562
neg_mtb_2702327	928.5939
pos_mtb_2841874	930.5646
pos_mtb_2559720	913.4666
neg_mtb_2668042	761.4934
pos_mtb_3431336	912.6564

pos_mtb_406044	236.0961
pos_mtb_3730327	751.6033
pos_mtb_2264430	551.2062
neg_mtb_2323000	500.225
neg_mtb_2129422	828.2434
neg_mtb_2959259	736.5558
neg_mtb_896187	523.0846
neg_mtb_2693416	916.6016
pos_mtb_2969615	632.4943
neg_mtb_2704304	481.4245
neg_mtb_2596037	905.5637
neg_mtb_679733	207.0525
pos_mtb_1963271	449.215
neg_mtb_2786576	769.5543
pos_mtb_2815525	941.4855
pos_mtb_547660	320.1157
pos_mtb_3198642	657.4976
neg_mtb_2423050	566.227
pos_mtb_2191827	333.2052
pos_mtb_1981286	341.2119
neg_mtb_2702375	945.6143
pos_mtb_2315585	854.5641
pos_mtb_3238514	806.6233
pos_mtb_2639443	763.5072
pos_mtb_2344266	807.5681
neg_mtb_2701593	805.5178
neg_mtb_2107444	313.1181
neg_mtb_2422031	420.252
neg_mtb_2722811	901.6038
neg_mtb_2693832	1007.625
neg_mtb_2773634	670.4864
pos_mtb_1879022	373.1978
neg_mtb_1054200	255.1069
neg_mtb_2430112	483.2254
neg_mtb_2608058	954.5653
pos_mtb_2157976	867.585
neg_mtb_2125745	433.2226
neg_mtb_2449227	203.181
neg_mtb_2458036	322.2462
neg_mtb_2714889	874.5777
neg_mtb_2286857	442.2273
neg_mtb_2683198	915.6007
pos_mtb_1617278	343.2251
neg_mtb_1169463	501.116
neg_mtb_2552596	856.5548
pos_mtb_2673822	963.4841
neg_mtb_2730130	689.4944
pos_mtb_3081251	446.3718
neg_mtb_2392523	484.205

neg_mtb_2707085	965.5853
pos_mtb_3178968	847.6261
pos_mtb_1878783	359.2182
neg_mtb_2700976	713.4756
pos_mtb_1801874	376.2161
neg_mtb_2391641	373.2028
pos_mtb_2206365	279.0846
pos_mtb_2639272	750.4744
neg_mtb_2688396	918.6064
pos_mtb_600444	310.127
neg_mtb_2436736	373.1942
pos_mtb_2078874	280.1548
neg_mtb_2663583	862.5655
pos_mtb_3043889	898.5256
pos_mtb_195782	261.1227
pos_mtb_2590841	960.465
pos_mtb_2946976	888.5714
pos_mtb_3373888	471.3719
neg_mtb_2683419	940.595
neg_mtb_2657559	928.6168
pos_mtb_547145	257.1243
pos_mtb_2310473	532.4354
pos_mtb_3084859	803.6057
neg_mtb_2286261	360.2251
pos_mtb_2914487	804.6129
pos_mtb_2588840	817.5775
pos_mtb_8036	168.0762
pos_mtb_1947054	965.4528
pos_mtb_2990011	818.5314
pos_mtb_2171670	261.1482
neg_mtb_2535027	295.2274
neg_mtb_2717508	611.4523
neg_mtb_2379444	375.2231
pos_mtb_3027318	964.4949
neg_mtb_2763918	633.4961
pos_mtb_2194196	583.2552
neg_mtb_2392284	460.227
pos_mtb_522620	295.0963
pos_mtb_868419	219.0824
neg_mtb_2737511	177.8443
pos_mtb_521976	166.0533
neg_mtb_2706254	814.5342
pos_mtb_2726689	794.5752
neg_mtb_2672777	730.5023
pos_mtb_2443169	860.5964
neg_mtb_2761649	962.6232
pos_mtb_2498503	779.5413
neg_mtb_2688148	883.646
pos_mtb_2239019	373.1982

pos_mtb_2873496	880.6338
pos_mtb_307520	277.1038
neg_mtb_2141566	434.2252
pos_mtb_2244753	813.5473
pos_mtb_2117304	851.5239
neg_mtb_2095554	843.2256
pos_mtb_3201219	896.5947
neg_mtb_2697883	904.5954
neg_mtb_2332785	355.1934
neg_mtb_2628970	933.6036
pos_mtb_2023706	862.5454
neg_mtb_2748834	692.515
neg_mtb_2711174	913.5823
neg_mtb_1693300	-
pos_mtb_3023993	658.511
pos_mtb_482260	291.1225
neg_mtb_2761580	942.595
neg_mtb_2749832	886.605
neg_mtb_2794318	768.552
neg_mtb_2710949	875.5779
pos_mtb_533607	243.1083
neg_mtb_2443874	501.2166
pos_mtb_2969917	666.5653
pos_mtb_305637	224.0764
neg_mtb_2740413	796.5449
pos_mtb_2558997	877.5261
neg_mtb_2682280	774.5273
neg_mtb_2672960	754.5027
neg_mtb_1441715	747.124
neg_mtb_2734306	643.4539
pos_mtb_3391696	854.6176
pos_mtb_2409035	710.4359
neg_mtb_2668730	866.5465
pos_mtb_3025774	827.5974
neg_mtb_2462422	918.5367
neg_mtb_2639314	894.5461
neg_mtb_2797909	695.5566
neg_mtb_2687238	770.5142
pos_mtb_587380	227.114
neg_mtb_2749375	791.5369
neg_mtb_2379273	357.213
neg_mtb_2707291	1014.564
neg_mtb_2764611	858.5773
neg_mtb_2730008	665.4547
pos_mtb_587381	228.1167
pos_mtb_2725288	688.4573
pos_mtb_6783706	911.7264
pos_mtb_3134272	862.6326
pos_mtb_2196761	875.5656

neg_mtb_2587654	786.4935
neg_mtb_2946112	735.5548
neg_mtb_2697591	850.5667
neg_mtb_2715001	894.535
pos_mtb_3134271	862.594
neg_mtb_2828843	446.3757
neg_mtb_2638784	828.5348
neg_mtb_2588903	925.5445
neg_mtb_2300469	415.2125
pos_mtb_5569238	776.5354
pos_mtb_2617338	976.5862
pos_mtb_2557293	792.566
pos_mtb_3064221	930.574
neg_mtb_2380524	499.2156
neg_mtb_2449742	293.2119
pos_mtb_2465257	722.4359
pos_mtb_2158294	893.5346
neg_mtb_2783149	912.6062
neg_mtb_2730011	666.4568
pos_mtb_2134467	674.4341
neg_mtb_2656477	788.5333
pos_mtb_3865125	445.3716
pos_mtb_432036	206.0862
pos_mtb_2244853	819.5962
pos_mtb_2501834	964.4667
pos_mtb_2838291	613.4824
neg_mtb_2478558	917.5353
neg_mtb_2706145	804.5176
neg_mtb_2710556	800.5428
neg_mtb_2770338	685.4769
neg_mtb_2697983	917.6038
pos_mtb_2395146	932.4935
pos_mtb_2838803	656.494
neg_mtb_2466133	341.2124
pos_mtb_1852578	399.2062
neg_mtb_2770302	671.4857
pos_mtb_2559400	897.5659
neg_mtb_2124614	313.1209
neg_mtb_2754040	881.5652
neg_mtb_2636661	527.1934
pos_mtb_2117471	867.5752
neg_mtb_2693013	849.567
neg_mtb_2710463	781.5181
pos_mtb_2500080	869.5449
pos_mtb_2587846	750.4647
neg_mtb_2674118	932.5857
neg_mtb_2745134	792.5617
neg_mtb_2682319	779.5059
neg_mtb_2449765	296.2313

neg_mtb_2664051	925.6058
pos_mtb_3043546	869.6053
pos_mtb_565909	262.124
neg_mtb_2687240	770.5357
pos_mtb_2246497	925.5566
pos_mtb_3134776	898.5926
neg_mtb_2633371	824.5438
neg_mtb_2565942	917.5355
pos_mtb_2758971	922.5438
neg_mtb_2781224	461.363
pos_mtb_2413105	920.5626
neg_mtb_2638134	740.5228
pos_mtb_566053	291.1184
pos_mtb_2990409	842.5871
pos_mtb_2468111	896.5856
neg_mtb_2403277	377.2321
neg_mtb_2777612	694.5335
pos_mtb_2501438	942.5446
pos_mtb_521887	149.0265
pos_mtb_3395705	641.5268
neg_mtb_777691	241.0311
neg_mtb_2764771	888.6169
neg_mtb_2682153	755.5056
neg_mtb_2673017	765.5344
pos_mtb_2315972	874.5144
neg_mtb_2734564	692.5059
neg_mtb_2688279	906.5756
neg_mtb_2143933	687.2339
neg_mtb_2260377	543.2246
neg_mtb_2450116	343.2312
pos_mtb_2289368	815.5576
neg_mtb_2595567	858.5331
pos_mtb_2726386	772.551
pos_mtb_2608557	319.2254
neg_mtb_2628229	815.5256
pos_mtb_2813994	849.6439
pos_mtb_3200156	794.5257
neg_mtb_2392276	459.2265
pos_mtb_2530465	891.5923
neg_mtb_2757053	716.5155
pos_mtb_2946101	837.6071
neg_mtb_2566011	928.5552
neg_mtb_2421374	301.2171
neg_mtb_2449752	295.2279
neg_mtb_2474163	345.2437
neg_mtb_2692284	760.5355
neg_mtb_2696869	757.515
pos_mtb_2090263	876.5245
pos_mtb_2559301	892.5845

pos_mtb_5907497	967.7158
neg_mtb_2258580	333.207
pos_mtb_2967630	430.3763
pos_mtb_470865	244.0941
neg_mtb_2752945	641.4668
pos_mtb_3373878	469.3651
neg_mtb_2763940	643.4732
pos_mtb_2557721	815.5625
neg_mtb_2764732	885.5969
pos_mtb_1808676	303.2321
neg_mtb_2678241	840.5679
neg_mtb_2682851	855.5555
pos_mtb_2946432	857.5071
pos_mtb_3042356	775.568
neg_mtb_2322485	425.2449
neg_mtb_2756951	691.4718
pos_mtb_2136955	871.5471
neg_mtb_2767163	667.5149
pos_mtb_2314536	802.5878
pos_mtb_1986120	796.535
neg_mtb_2761491	914.5765
neg_mtb_2478692	933.5344
pos_mtb_6329059	926.7133
pos_mtb_2991597	922.5333
neg_mtb_2616949	770.4966
neg_mtb_2449746	294.2152
pos_mtb_5942969	935.7327

54 metabolites

neg_mtb_1076610	245.9123
neg_mtb_1055224	309.8757
neg_mtb_5972	332.0158
neg_mtb_2936494	765.0338
neg_mtb_1054079	247.9067
neg_mtb_2922	175.0187
neg_mtb_1076668	248.9023
neg_mtb_649598	594.1673
neg_mtb_5606	308.0243
pos_mtb_3869597	746.5739
neg_mtb_585131	388.0735
pos_mtb_1907626	319.305
neg_mtb_646281	220.9753
neg_mtb_2616158	705.0659
neg_mtb_2770715	810.525
neg_mtb_7680	423.2667
neg_mtb_710399	301.8857
neg_mtb_910338	337.0143
neg_mtb_5622	309.0224
neg_mtb_3375	202.0136

neg_mtb_3226087	175.0179
neg_mtb_2735236	791.5049
neg_mtb_6014	335.0089
neg_mtb_633108	794.1973
neg_mtb_30334	146.9691
neg_mtb_646048	202.0256
neg_mtb_607534	474.0316
neg_mtb_31369	303.0341
neg_mtb_1227	99.9713
neg_mtb_651014	769.198
neg_mtb_694923	178.033
neg_mtb_4868	273.004
neg_mtb_668115	743.2032
neg_mtb_1054029	244.9109
neg_mtb_1162	96.9749
neg_mtb_5996	334.0125
neg_mtb_4523	261.024
neg_mtb_757598	184.0971
neg_mtb_630869	498.0275
neg_mtb_929706	501.0383
neg_mtb_649252	550.1259
neg_mtb_3857	222.0037
neg_mtb_27540	154.9465
neg_mtb_496211	223.9942
neg_mtb_1055179	307.8768
neg_mtb_649339	559.1118
neg_mtb_5113	290.0124
neg_mtb_4722	264.0136
neg_mtb_2809326	562.4536
neg_mtb_2845	172.0019
neg_mtb_2231069	371.056
neg_mtb_608211	551.1174
neg_mtb_9779	526.3243
neg_mtb_645132	124.0388

31 metabolites

pos_mtb_3869597	746.5739
pos_mtb_3440744	721.5569
neg_mtb_2770715	810.525
pos_mtb_3429119	720.5544
neg_mtb_757598	184.0971
neg_mtb_1076668	248.9023
neg_mtb_2675721	313.3019
pos_mtb_2970857	772.5034
neg_mtb_2743494	441.3846
neg_mtb_2508586	328.2921
neg_mtb_2766492	454.3962
pos_mtb_470729	132.0567
pos_mtb_2702980	849.5356

pos_mtb_2786284	837.4861
neg_mtb_1054079	247.9067
pos_mtb_2756896	791.517
pos_mtb_2442998	846.5254
pos_mtb_4022003	799.6044
pos_mtb_4074974	798.6026
neg_mtb_2730276	720.5258
neg_mtb_476314	337.0521
pos_mtb_3960979	800.6071
pos_mtb_3349292	796.5175
pos_mtb_2785269	759.4942
pos_mtb_2725492	709.5226
pos_mtb_2786846	878.4849
neg_mtb_2742777	203.0361
neg_mtb_2868434	924.5927
neg_mtb_2865421	925.5958
pos_mtb_3200775	851.5447
pos_mtb_3200763	850.5425

76 metabolites

neg_mtb_2378454	253.0838
neg_mtb_2365047	254.0866
pos_mtb_2192576	415.2543
neg_mtb_860573	175.0146
pos_mtb_1940995	415.2078
pos_mtb_1947041	964.4481
neg_mtb_2668004	754.4964
neg_mtb_2568345	317.2477
pos_mtb_470827	205.0816
neg_mtb_2718815	880.5712
neg_mtb_2761177	856.5713
pos_mtb_2971442	823.5659
pos_mtb_2990081	822.5621
neg_mtb_2767708	857.573
pos_mtb_3042910	821.5486
pos_mtb_2913737	756.5535
pos_mtb_3273976	848.5756
pos_mtb_3009920	820.5463
pos_mtb_2557521	804.5533
pos_mtb_3025995	844.5455
pos_mtb_2971161	799.5671
neg_mtb_2828847	447.3775
pos_mtb_3179244	873.5771
neg_mtb_2702110	884.5844
pos_mtb_3178967	847.5563
pos_mtb_2970668	757.5566
pos_mtb_3376556	849.5782
pos_mtb_3200711	846.5569
pos_mtb_2915004	836.5563

pos_mtb_3701041	833.6365
neg_mtb_2720535	388.3317
neg_mtb_2748055	510.4627
neg_mtb_2458019	319.2278
neg_mtb_2249100	918.3932
neg_mtb_2563115	570.4771
neg_mtb_2906452	218.0312
neg_mtb_2654002	497.421
pos_mtb_3701021	832.6331
pos_mtb_2207604	415.3448
neg_mtb_2708600	387.326
neg_mtb_2676494	527.4543
neg_mtb_2636475	498.4223
pos_mtb_2171178	185.1317
pos_mtb_2608525	316.1939
neg_mtb_2812564	419.3877
neg_mtb_2766753	536.4767
neg_mtb_2903463	217.0345
neg_mtb_3220231	223.0202
neg_mtb_2789095	417.3729
neg_mtb_2712773	388.3276
neg_mtb_2738919	535.4658
neg_mtb_2720807	453.4318
pos_mtb_2222701	485.3476
neg_mtb_2568256	304.0525
pos_mtb_2220886	318.2875
pos_mtb_2749937	318.2851
neg_mtb_716877	947.3049
pos_mtb_2037957	440.2259
pos_mtb_1653168	318.2892
neg_mtb_2523412	498.4236
neg_mtb_2647866	526.3946
neg_mtb_2681333	623.3138
neg_mtb_2682645	818.575
neg_mtb_2549447	454.1952
neg_mtb_2129579	849.2234
neg_mtb_2492407	447.5009
neg_mtb_2555828	455.1935
neg_mtb_2672935	749.527
pos_mtb_2039289	530.3627
neg_mtb_2245521	521.1948
neg_mtb_2610920	646.4252
pos_mtb_3458152	797.5308
neg_mtb_716740	930.3054
neg_mtb_2761294	877.4153
neg_mtb_2619474	101.933
neg_mtb_2660460	443.2791

33 metabolites

pos_mtb_1947041	964.4481
neg_mtb_2568345	317.2477
pos_mtb_470827	205.0816
neg_mtb_2718815	880.5712
neg_mtb_2761177	856.5713
pos_mtb_2971442	823.5659
pos_mtb_2990081	822.5621
neg_mtb_2767708	857.573
pos_mtb_3042910	821.5486
pos_mtb_2913737	756.5535
pos_mtb_3273976	848.5756
pos_mtb_3009920	820.5463
pos_mtb_2557521	804.5533
pos_mtb_3025995	844.5455
pos_mtb_2971161	799.5671
neg_mtb_2828847	447.3775
pos_mtb_3179244	873.5771
neg_mtb_2702110	884.5844
pos_mtb_3178967	847.5563
pos_mtb_2970668	757.5566
pos_mtb_3376556	849.5782
pos_mtb_3200711	846.5569
pos_mtb_2915004	836.5563
pos_mtb_3701041	833.6365
neg_mtb_2458019	319.2278
pos_mtb_3701021	832.6331
neg_mtb_2708600	387.326
neg_mtb_2712773	388.3276
neg_mtb_757598	184.0971
neg_mtb_2770715	810.525
pos_mtb_3869597	746.5739
neg_mtb_1076668	248.9023
neg_mtb_1054079	247.9067

604 metabolites

rLC_pos_mtb_666808	278.2144
rLC_pos_mtb_164348	178.003
rLC_pos_mtb_1996309	517.9946
rLC_pos_mtb_1871599	294.0582
rLC_pos_mtb_883502	280.105
rLC_pos_mtb_411077	311.0041
rLC_pos_mtb_2869365	529.3661
rLC_pos_mtb_867216	1330.006
rLC_pos_mtb_164191	153.9967
rLC_pos_mtb_155412	261.0363
rLC_pos_mtb_2132543	305.1746
rLC_pos_mtb_1898669	352.0936
rLC_pos_mtb_1237119	221.0645

rLC_pos_mtb_164430	244.0315
rLC_pos_mtb_1675929	245.0058
rLC_pos_mtb_2066918	301.2518
rLC_pos_mtb_162660	382.0979
rLC_pos_mtb_608400	579.1522
rLC_pos_mtb_407845	409.0844
rLC_pos_mtb_166131	235.0217
rLC_pos_mtb_893107	524.0248
rLC_pos_mtb_879807	986.0247
rLC_pos_mtb_727999	182.1146
rLC_pos_mtb_2075071	344.3465
rLC_pos_mtb_930987	413.0974
rLC_pos_mtb_164571	301.0529
rLC_pos_mtb_865937	922.0065
rLC_pos_mtb_887199	1149.024
rLC_pos_mtb_886307	898.0051
rLC_pos_mtb_497874	68.0493
rLC_pos_mtb_2116346	252.2041
rLC_pos_mtb_303482	400.1455
rLC_pos_mtb_1975972	235.2047
rLC_pos_mtb_1922108	633.2844
rLC_pos_mtb_2040101	345.2554
rLC_pos_mtb_874249	1329.014
rLC_pos_mtb_1921153	538.4559
rLC_pos_mtb_449599	283.0421
rLC_pos_mtb_1921544	579.8449
rLC_pos_mtb_899665	138.0736
rLC_pos_mtb_1899531	458.6245
rLC_pos_mtb_1921964	617.2621
rLC_pos_mtb_607112	161.0825
rLC_pos_mtb_877667	413.0075
rLC_pos_mtb_2088520	306.1357
rLC_pos_mtb_1901159	617.2582
rLC_pos_mtb_879127	822.0237
rLC_pos_mtb_15660	284.0643
rLC_pos_mtb_162505	185.9892
rLC_pos_mtb_1951969	346.1335
rLC_pos_mtb_1898119	233.1944
rLC_pos_mtb_164368	195.0257
rLC_pos_mtb_832381	1458.035
rLC_pos_mtb_1919761	314.091
rLC_pos_mtb_1951772	272.0772
rLC_pos_mtb_1920196	410.4685
rLC_pos_mtb_2077750	601.2654
rLC_pos_mtb_164697	372.0725
rLC_pos_mtb_877664	412.0129
rLC_pos_mtb_1047867	187.0573
rLC_pos_mtb_1960779	777.9746
rLC_pos_mtb_830918	1200.012

rLC_pos_mtb_879773	979.0043
rLC_pos_mtb_1899564	461.1361
rLC_pos_mtb_878616	658.0223
rLC_pos_mtb_1900771	588.2771
rLC_pos_mtb_579601	134.0785
rLC_pos_mtb_841838	1581.043
rLC_pos_mtb_878853	740.0229
rLC_pos_mtb_6090434	160.1272
rLC_pos_mtb_590241	150.0758
rLC_pos_mtb_2277763	543.1266
rLC_pos_mtb_1366511	247.0423
rLC_pos_mtb_877397	331.0058
rLC_pos_mtb_872988	1002.006
rLC_pos_mtb_1952946	599.2521
rLC_pos_mtb_878297	576.0167
rLC_pos_mtb_839752	1376.034
rLC_pos_mtb_175273	164.0082
rLC_pos_mtb_1965593	140.0163
rLC_pos_mtb_1847460	372.0665
rLC_pos_mtb_838817	1253.533
rLC_pos_mtb_634144	244.1071
rLC_pos_mtb_1898739	367.0625
rLC_pos_mtb_1447092	104.9924
rLC_pos_mtb_830487	1048.526
rLC_pos_mtb_830728	1130.532
rLC_pos_mtb_1967379	533.1957
rLC_pos_mtb_1936533	181.0482
rLC_pos_mtb_155441	284.0532
rLC_pos_mtb_1951986	359.2972
rLC_pos_mtb_318845	589.1464
rLC_pos_mtb_1003886	308.9653
rLC_pos_mtb_885687	734.9528
rLC_pos_mtb_879797	985.0242
rLC_pos_mtb_1952034	380.0952
rLC_pos_mtb_879442	897.0062
rLC_pos_mtb_634135	241.0317
rLC_pos_mtb_885461	652.9525
rLC_pos_mtb_902974	472.9725
rLC_pos_mtb_2029126	471.343
rLC_pos_mtb_873734	1165.013
rLC_pos_mtb_1960232	663.4518
rLC_pos_mtb_1977217	402.2148
rLC_pos_mtb_1967004	465.2216
rLC_pos_mtb_1899914	494.1846
rLC_pos_mtb_872159	838.0038
rLC_pos_mtb_877927	495.013
rLC_pos_mtb_2548492	777.5914
rLC_pos_mtb_1936977	303.1993
rLC_pos_mtb_871839	756.0016

rLC_pos_mtb_163729	74.0238
rLC_pos_mtb_1939020	586.2828
rLC_pos_mtb_1787724	239.1237
rLC_pos_mtb_1996714	559.2985
rLC_pos_mtb_885681	733.0045
rLC_pos_mtb_893096	523.0244
rLC_pos_mtb_838615	1212.032
rLC_pos_mtb_163914	122.027
rLC_pos_mtb_838818	1254.034
rLC_pos_mtb_830724	1130.026
rLC_pos_mtb_407855	485.1364
rLC_pos_mtb_872339	868.9515
rLC_pos_mtb_873403	1083.011
rLC_pos_mtb_830486	1048.024
rLC_pos_mtb_877926	494.0153
rLC_pos_mtb_2041415	464.2169
rLC_pos_mtb_1944036	1093.674
rLC_pos_mtb_1875373	602.2657
rLC_pos_mtb_878336	591.9957
rLC_pos_mtb_2897740	552.427
rLC_pos_mtb_2353850	309.0285
rLC_pos_mtb_2028422	387.3259
rLC_pos_mtb_666806	277.2125
rLC_pos_mtb_173669	142.0263
rLC_pos_mtb_2039478	255.1227
rLC_pos_mtb_879119	821.0232
rLC_pos_mtb_1936625	202.1128
rLC_pos_mtb_893646	570.947
rLC_pos_mtb_634044	177.1044
rLC_pos_mtb_1919904	351.0965
rLC_pos_mtb_891106	294.9283
rLC_pos_mtb_1919560	230.185
rLC_pos_mtb_884097	359.0172
rLC_pos_mtb_163909	120.0113
rLC_pos_mtb_840109	1417.041
rLC_pos_mtb_1047815	184.0942
rLC_pos_mtb_2100310	251.2009
rLC_pos_mtb_607682	386.1465
rLC_pos_mtb_1938263	509.1765
rLC_pos_mtb_1761587	341.1758
rLC_pos_mtb_657170	134.0813
rLC_pos_mtb_1958811	327.2263
rLC_pos_mtb_1966216	328.1026
rLC_pos_mtb_1421635	268.9987
rLC_pos_mtb_1921134	536.186
rLC_pos_mtb_666863	303.2268
rLC_pos_mtb_878845	739.0216
rLC_pos_mtb_1951962	343.286
rLC_pos_mtb_2074718	293.0565

rLC_pos_mtb_1821107	202.1184
rLC_pos_mtb_1849830	576.2627
rLC_pos_mtb_884293	411.0116
rLC_pos_mtb_162465	151.9834
rLC_pos_mtb_878295	575.0163
rLC_pos_mtb_1965966	261.1446
rLC_pos_mtb_3047567	823.5438
rLC_pos_mtb_2153003	506.4277
rLC_pos_mtb_162541	242.0339
rLC_pos_mtb_164570	300.0508
rLC_pos_mtb_162543	243.028
rLC_pos_mtb_1097345	261.0583
rLC_pos_mtb_607105	160.0786
rLC_pos_mtb_1996436	532.2035
rLC_pos_mtb_164568	299.0552
rLC_pos_mtb_1966998	464.2172
rLC_pos_mtb_1976683	345.1292
rLC_pos_mtb_1941308	852.4862
rLC_pos_mtb_666606	161.1363
rLC_pos_mtb_645135	391.1024
rLC_pos_mtb_884223	390.968
rLC_pos_mtb_1951771	271.0748
rLC_pos_mtb_1608535	229.0319
rLC_pos_mtb_681165	202.1188
rLC_pos_mtb_2897737	551.4244
rLC_pos_mtb_1919955	364.2651
rLC_pos_mtb_2703278	779.5864
rLC_pos_mtb_408712	135.0624
rLC_pos_mtb_870199	329.0078
rLC_pos_mtb_1939169	601.265
rLC_pos_mtb_1872380	358.2942
rLC_pos_mtb_1965621	152.0541
rLC_pos_mtb_1951776	275.111
rLC_pos_mtb_2014749	558.296
rLC_pos_mtb_2011560	185.1289
rLC_pos_mtb_1937296	366.0586
rLC_pos_mtb_879190	837.0018
rLC_pos_mtb_911123	137.071
rLC_pos_mtb_1965956	256.1758
rLC_pos_mtb_877921	493.0133
rLC_pos_mtb_878159	540.9379
rLC_pos_mtb_3047559	822.5419
rLC_pos_mtb_1994905	386.3257
rLC_pos_mtb_164692	370.074
rLC_pos_mtb_590087	107.0717
rLC_pos_mtb_878897	755.0009
rLC_pos_mtb_579830	203.0529
rLC_pos_mtb_1849037	478.3126
rLC_pos_mtb_878645	672.9955

rLC_pos_mtb_2058856	505.425
rLC_pos_mtb_884161	376.9328
rLC_pos_mtb_1966153	314.0823
rLC_pos_mtb_1937275	363.1635
rLC_pos_mtb_878334	590.9936
rLC_pos_mtb_652157	113.071
rLC_pos_mtb_681144	186.1124
rLC_pos_mtb_2088289	261.1483
rLC_pos_mtb_877146	299.0089
rLC_pos_mtb_1958835	335.0626
rLC_pos_mtb_884707	508.9911
rLC_pos_mtb_1720800	160.1331
rLC_pos_mtb_1872146	342.283
rLC_pos_mtb_634037	176.1031
rLC_pos_mtb_1419965	140.0683
rLC_pos_mtb_1953458	731.9585
rLC_pos_mtb_164567	298.0535
rLC_pos_mtb_162539	241.0318
rLC_pos_mtb_1937204	349.1841
rLC_pos_mtb_579576	132.0658
rLC_pos_mtb_1936983	305.1578
rLC_pos_mtb_2012230	302.1969
rLC_pos_mtb_662590	203.0529
rLC_pos_mtb_652205	159.0768
rLC_pos_mtb_662561	160.1335
rLC_pos_mtb_493034	154.0873
rLC_pos_mtb_1966151	313.0809
rLC_pos_mtb_1872145	341.2808
rLC_pos_mtb_1958809	327.2026
rLC_pos_mtb_1966111	300.2028
rLC_pos_mtb_2506410	152.071
rLC_pos_mtb_1965906	239.1497
rLC_pos_mtb_1958613	239.1504
rLC_pos_mtb_1976243	283.1762
rLC_pos_mtb_282474	136.0642
rLC_pos_mtb_1966780	432.2813
rLC_pos_mtb_1966295	344.2289
rLC_pos_mtb_1994924	388.2554
rLC_neg_mtb_2163430	1535.566
rLC_neg_mtb_2183999	1128.056
rLC_neg_mtb_2161864	1453.565
rLC_neg_mtb_1602641	1076.065
rLC_neg_mtb_1677403	1537.056
rLC_neg_mtb_1339538	753.2771
rLC_neg_mtb_2188920	1412.565
rLC_neg_mtb_1321914	734.1956
rLC_neg_mtb_1331853	151.9842
rLC_neg_mtb_1369731	589.338
rLC_neg_mtb_2895705	883.9851

rLC_neg_mtb_1731829	1676.027
rLC_neg_mtb_2175561	594.296
rLC_neg_mtb_1292957	795.1953
rLC_neg_mtb_2869766	1046.987
rLC_neg_mtb_1700202	1350.016
rLC_neg_mtb_1679190	1652.985
rLC_neg_mtb_1274555	803.1848
rLC_neg_mtb_1725345	1268.015
rLC_neg_mtb_2161894	1455.066
rLC_neg_mtb_1649539	1487.984
rLC_neg_mtb_3374368	618.9376
rLC_neg_mtb_1603925	1186.008
rLC_neg_mtb_1651484	1650.986
rLC_neg_mtb_2164556	1594.032
rLC_neg_mtb_1900163	1595.024
rLC_neg_mtb_2161102	1412.064
rLC_neg_mtb_2123940	964.0535
rLC_neg_mtb_2433714	1150.983
rLC_neg_mtb_2170047	248.9016
rLC_neg_mtb_1579641	631.0544
rLC_neg_mtb_3030844	824.9672
rLC_neg_mtb_1274549	802.1855
rLC_neg_mtb_1815901	1594.026
rLC_neg_mtb_1638214	631.9979
rLC_neg_mtb_3061023	876.9653
rLC_neg_mtb_1626004	1323.975
rLC_neg_mtb_2536619	823.9744
rLC_neg_mtb_2972976	638.9752
rLC_neg_mtb_2999152	555.9745
rLC_neg_mtb_1588521	1568.978
rLC_neg_mtb_1809237	1186.013
rLC_neg_mtb_1292855	778.1915
rLC_neg_mtb_2160411	1373.065
rLC_neg_mtb_1582366	915.9569
rLC_neg_mtb_1623287	1104.008
rLC_neg_mtb_1931711	1513.023
rLC_neg_mtb_1992077	1512.024
rLC_neg_mtb_2091291	700.9481
rLC_neg_mtb_1584522	1159.967
rLC_neg_mtb_1677904	1568.984
rLC_neg_mtb_1643585	1022.005
rLC_neg_mtb_1627965	1488.976
rLC_neg_mtb_1731810	1675.025
rLC_neg_mtb_2533493	660.9654
rLC_neg_mtb_2489107	1068.977
rLC_neg_mtb_1489719	165.042
rLC_neg_mtb_2972953	637.9771
rLC_neg_mtb_1607341	1486.977
rLC_neg_mtb_1602665	1077.966

rLC_neg_mtb_1254553	243.9915
rLC_neg_mtb_1648507	1406.974
rLC_neg_mtb_2839579	964.9851
rLC_neg_mtb_2457443	973.0043
rLC_neg_mtb_3157256	630.957
rLC_neg_mtb_2313857	566.9225
rLC_neg_mtb_1292837	775.1924
rLC_neg_mtb_1990551	1430.022
rLC_neg_mtb_2314190	591.0018
rLC_neg_mtb_2512592	988.9753
rLC_neg_mtb_1675724	1431.017
rLC_neg_mtb_2535019	741.9724
rLC_neg_mtb_2028753	511.2909
rLC_neg_mtb_2973216	652.9475
rLC_neg_mtb_1527282	391.0035
rLC_neg_mtb_2924254	816.9539
rLC_neg_mtb_1350294	270.9451
rLC_neg_mtb_3059685	794.9633
rLC_neg_mtb_1626993	1404.976
rLC_neg_mtb_1560258	337.0141
rLC_neg_mtb_1292068	614.1364
rLC_neg_mtb_2165033	1618.068
rLC_neg_mtb_2186778	1291.063
rLC_neg_mtb_4622170	317.2476
rLC_neg_mtb_1273366	615.1349
rLC_neg_mtb_3028138	672.9971
rLC_neg_mtb_1698130	1209.057
rLC_neg_mtb_1642552	940.0028
rLC_neg_mtb_3904414	477.2668
rLC_neg_mtb_1700189	1349.015
rLC_neg_mtb_1304376	358.1039
rLC_neg_mtb_1490118	224.0854
rLC_neg_mtb_1338012	599.2866
rLC_neg_mtb_2457665	986.9758
rLC_neg_mtb_1223196	513.1559
rLC_neg_mtb_3958871	229.537
rLC_neg_mtb_1993671	1593.025
rLC_neg_mtb_1315857	138.0016
rLC_neg_mtb_1350332	272.9433
rLC_neg_mtb_2485597	906.9742
rLC_neg_mtb_1237743	284.992
rLC_neg_mtb_2479409	591.2766
rLC_neg_mtb_1641381	858.0014
rLC_neg_mtb_1674514	1348.017
rLC_neg_mtb_1624962	1242.966
rLC_neg_mtb_1723116	1127.056
rLC_neg_mtb_1673255	1266.015
rLC_neg_mtb_1625994	1322.974
rLC_neg_mtb_1713910	571.0468

rLC_neg_mtb_1663958	718.045
rLC_neg_mtb_1542444	212.0577
rLC_neg_mtb_1369286	547.1671
rLC_neg_mtb_2163439	1536.068
rLC_neg_mtb_1725331	1267.012
rLC_neg_mtb_2061027	593.2928
rLC_neg_mtb_2105566	1511.022
rLC_neg_mtb_1619453	775.9971
rLC_neg_mtb_1671989	1184.013
rLC_neg_mtb_1623950	1160.964
rLC_neg_mtb_2753268	799.0447
rLC_neg_mtb_1712557	489.0457
rLC_neg_mtb_2704737	1044.052
rLC_neg_mtb_2645075	561.9921
rLC_neg_mtb_2161875	1454.066
rLC_neg_mtb_2455195	824.9727
rLC_neg_mtb_1292172	632.1469
rLC_neg_mtb_1585339	1240.968
rLC_neg_mtb_1292817	769.2021
rLC_neg_mtb_2169972	244.9073
rLC_neg_mtb_2139714	246.9046
rLC_neg_mtb_2535687	774.9938
rLC_neg_mtb_4413261	183.1385
rLC_neg_mtb_2782384	808.9967
rLC_neg_mtb_2589119	480.9855
rLC_neg_mtb_2395946	742.972
rLC_neg_mtb_1624238	1185.008
rLC_neg_mtb_1670699	1102.011
rLC_neg_mtb_2456368	904.9733
rLC_neg_mtb_1292847	777.1865
rLC_neg_mtb_3181081	496.2978
rLC_neg_mtb_1337345	540.1569
rLC_neg_mtb_1292843	776.188
rLC_neg_mtb_2564077	643.9949
rLC_neg_mtb_1603595	1158.967
rLC_neg_mtb_3606986	396.9827
rLC_neg_mtb_1223011	472.0885
rLC_neg_mtb_2534738	726.9954
rLC_neg_mtb_1331542	120.0122
rLC_neg_mtb_1675695	1429.018
rLC_neg_mtb_4392930	505.3133
rLC_neg_mtb_1601884	996.9578
rLC_neg_mtb_5036166	374.316
rLC_neg_mtb_1664382	740.9722
rLC_neg_mtb_1721807	1045.054
rLC_neg_mtb_4094308	211.1692
rLC_neg_mtb_2749720	562.9885
rLC_neg_mtb_1643563	1020.008
rLC_neg_mtb_2192403	1617.068

rLC_neg_mtb_2923982	800.9786
rLC_neg_mtb_1644593	1103.006
rLC_neg_mtb_3942785	492.2864
rLC_neg_mtb_1939482	298.9482
rLC_neg_mtb_1351528	357.0976
rLC_neg_mtb_2760450	1207.054
rLC_neg_mtb_2160394	1372.064
rLC_neg_mtb_2483864	822.9717
rLC_neg_mtb_1674502	1347.016
rLC_neg_mtb_1337322	538.1588
rLC_neg_mtb_2780772	717.0421
rLC_neg_mtb_1351427	352.1124
rLC_neg_mtb_3105225	577.2975
rLC_neg_mtb_1642525	938.0046
rLC_neg_mtb_2067234	963.0516
rLC_neg_mtb_1622036	994.9619
rLC_neg_mtb_2974641	740.9712
rLC_neg_mtb_2186758	1290.062
rLC_neg_mtb_1383302	372.0754
rLC_neg_mtb_1621079	914.9558
rLC_neg_mtb_2511271	889.0006
rLC_neg_mtb_1978329	806.9971
rLC_neg_mtb_2832112	553.0361
rLC_neg_mtb_2163420	1535.067
rLC_neg_mtb_1669448	1021.003
rLC_neg_mtb_1368247	460.0953
rLC_neg_mtb_1641355	856.002
rLC_neg_mtb_1698944	1265.013
rLC_neg_mtb_3941446	328.1626
rLC_neg_mtb_2837837	880.046
rLC_neg_mtb_1711115	407.043
rLC_neg_mtb_1332248	183.0315
rLC_neg_mtb_2590670	576.9623
rLC_neg_mtb_1857706	701.0153
rLC_neg_mtb_2122555	881.0486
rLC_neg_mtb_2892492	718.9769
rLC_neg_mtb_2535002	740.9677
rLC_neg_mtb_4253101	600.3313
rLC_neg_mtb_1290543	410.1157
rLC_neg_mtb_1349627	200.0408
rLC_neg_mtb_1582342	912.9575
rLC_neg_mtb_1335884	410.041
rLC_neg_mtb_2157402	1208.057
rLC_neg_mtb_2921564	636.9741
rLC_neg_mtb_2695196	494.9593
rLC_neg_mtb_1640178	773.9991
rLC_neg_mtb_1668044	939.0009
rLC_neg_mtb_2508501	658.9651
rLC_neg_mtb_4507888	242.2204

rLC_neg_mtb_1292178	633.144
rLC_neg_mtb_1306254	588.1414
rLC_neg_mtb_1631644	175.0244
rLC_neg_mtb_1366626	304.054
rLC_neg_mtb_1697740	1183.011
rLC_neg_mtb_2999137	554.9717
rLC_neg_mtb_1380520	83.0139
rLC_neg_mtb_2536273	806.9966
rLC_neg_mtb_5370027	440.3819
rLC_neg_mtb_2161853	1453.065
rLC_neg_mtb_1367793	426.1126
rLC_neg_mtb_2782192	798.0435
rLC_neg_mtb_1367078	350.1131
rLC_neg_mtb_2126605	1126.056
rLC_neg_mtb_4008619	183.1385
rLC_neg_mtb_1291916	589.1377
rLC_neg_mtb_4009316	229.0352
rLC_neg_mtb_1641367	856.9974
rLC_neg_mtb_2064457	799.0455
rLC_neg_mtb_4489668	605.3651
rLC_neg_mtb_1946240	724.994
rLC_neg_mtb_1670689	1101.007
rLC_neg_mtb_4276022	414.3288
rLC_neg_mtb_1221221	123.0323
rLC_neg_mtb_3962113	491.2835
rLC_neg_mtb_3962141	493.2969
rLC_neg_mtb_1367161	357.1017
rLC_neg_mtb_2160377	1371.062
rLC_neg_mtb_1331709	140.0114
rLC_neg_mtb_3181072	495.2946
rLC_neg_mtb_1337336	539.158
rLC_neg_mtb_1944946	642.9916
rLC_neg_mtb_1291908	587.1434
rLC_neg_mtb_1857552	692.9917
rLC_neg_mtb_1640196	774.9944
rLC_neg_mtb_2888895	498.9286
rLC_neg_mtb_3206442	498.9309
rLC_neg_mtb_2565462	724.9943
rLC_neg_mtb_2097417	1044.053
rLC_neg_mtb_1221168	108.0439
rLC_neg_mtb_1380830	112.9876
rLC_neg_mtb_1715023	635.0404
rLC_neg_mtb_1366603	302.0643
rLC_neg_mtb_1669410	1019.005
rLC_neg_mtb_2807536	690.9932
rLC_neg_mtb_1271588	436.1125
rLC_neg_mtb_1352818	461.0924
rLC_neg_mtb_4465734	504.3087
rLC_neg_mtb_1273301	607.1478

rLC_neg_mtb_2288235	635.0396
rLC_neg_mtb_1238905	430.0807
rLC_neg_mtb_4322578	327.2888
rLC_neg_mtb_2129295	1289.058
rLC_neg_mtb_2063057	717.0429
rLC_neg_mtb_2829499	396.9824
rLC_neg_mtb_2645058	560.9883
rLC_neg_mtb_1271354	419.0985
rLC_neg_mtb_1398565	223.0821
rLC_neg_mtb_1368222	458.1414
rLC_neg_mtb_2037671	962.0507
rLC_neg_mtb_1306991	741.2068
rLC_neg_mtb_1203009	431.076
rLC_neg_mtb_2811401	961.0462
rLC_neg_mtb_2834744	716.041
rLC_neg_mtb_1639031	692.9915
rLC_neg_mtb_2591741	642.9913
rLC_neg_mtb_1337087	515.1411
rLC_neg_mtb_1306204	580.1544
rLC_neg_mtb_1350022	239.0161
rLC_neg_mtb_1290493	406.0145
rLC_neg_mtb_1668005	937.0022
rLC_neg_mtb_1130322	408.0126
rLC_neg_mtb_1365585	228.9935
rLC_neg_mtb_1333772	296.0373
rLC_neg_mtb_2127967	1207.056
rLC_neg_mtb_2065844	880.0471
rLC_neg_mtb_2088627	553.0369
rLC_neg_mtb_1398763	248.006
rLC_neg_mtb_1182141	328.0876
rLC_neg_mtb_1574833	199.0588
rLC_neg_mtb_1202987	428.0823
rLC_neg_mtb_1201642	200.0247
rLC_neg_mtb_1641338	854.9994
rLC_neg_mtb_2748496	478.9855
rLC_neg_mtb_4466711	579.3492
rLC_neg_mtb_1292027	606.1519
rLC_neg_mtb_1306213	581.1516
rLC_neg_mtb_5197418	439.3776
rLC_neg_mtb_1365017	180.0658
rLC_neg_mtb_1335672	395.101
rLC_neg_mtb_2034521	798.0446
rLC_neg_mtb_4489664	604.361
rLC_neg_mtb_2098874	1125.053
rLC_neg_mtb_1239231	468.0953
rLC_neg_mtb_1367090	351.1102
rLC_neg_mtb_1365262	199.0374
rLC_neg_mtb_1256634	510.0967
rLC_neg_mtb_1640163	772.9967

rLC_neg_mtb_1331962	165.0412
rLC_neg_mtb_1380774	107.0375
rLC_neg_mtb_4390012	241.2166
rLC_neg_mtb_1367782	425.1091
rLC_neg_mtb_4276001	413.3261
rLC_neg_mtb_4441255	478.2934
rLC_neg_mtb_1352384	423.1088
rLC_neg_mtb_1350376	274.0466
rLC_neg_mtb_1305300	454.1248
rLC_neg_mtb_1316291	182.0277
rLC_neg_mtb_4392464	452.2781
rLC_neg_mtb_2068514	1043.051
rLC_neg_mtb_1305023	429.0788
rLC_neg_mtb_1382700	303.0534
rLC_neg_mtb_1638998	690.9938
rLC_neg_mtb_1271598	437.1085
rLC_neg_mtb_1617363	608.9908
rLC_neg_mtb_2058959	470.0327
rLC_neg_mtb_2029571	552.0356
rLC_neg_mtb_2031239	634.0389
rLC_neg_mtb_1490269	247.0115
rLC_neg_mtb_2032876	716.0418
rLC_neg_mtb_2026223	388.0303
rLC_neg_mtb_1254832	276.0004
rLC_neg_mtb_1435521	142.9981
rLC_neg_mtb_1365564	226.996
rLC_neg_mtb_1335709	398.0414
rLC_neg_mtb_1337072	514.1371
rLC_neg_mtb_1366666	311.0714
rLC_neg_mtb_1270739	356.0983
rLC_neg_mtb_2067206	961.0482
rLC_neg_mtb_1292017	605.1533
rLC_neg_mtb_1320305	526.1011
rLC_neg_mtb_1331971	166.0489
rLC_neg_mtb_4393576	578.346
rLC_neg_mtb_2036081	879.0454
rLC_neg_mtb_1436551	272.0463
rLC_neg_mtb_2647640	715.0387
rLC_neg_mtb_1287799	115.0038
rLC_neg_mtb_2034507	797.0422
rLC_neg_mtb_1318069	327.0915
rLC_neg_mtb_1302474	150.0561
rLC_neg_mtb_1383724	424.114
rLC_neg_mtb_2032862	715.0394
rLC_neg_mtb_1288997	260.0237
rLC_neg_mtb_2004334	633.0364
rLC_neg_mtb_2003108	551.0333
rLC_neg_mtb_2001925	469.0303
rLC_neg_mtb_1382124	246.0073

rLC_neg_mtb_1350333	273.0433
rLC_neg_mtb_1351098	326.0882
rLC_neg_mtb_1268940	230.0135

Scientific Advisory Committee

M. Katz (Chair), M.K. Bhan, C. Garza, A. Langer, P.M. Rothwell, S. Zaidi.

Steering Committee

R. Uauy (Chair), S.H. Kennedy (Co-Principal Investigator), J. Villar (Co-Principal Investigator), D.G. Altman (died 2018), F.C. Barros, J.A. Berkley, F. Burton, M. Carvalho, L. Cheikh Ismail, W.C. Chumlea, A. Lambert, S. Munim, S. Norris, F. Nosten, A.T. Papageorghiou, C. Victora.

Executive Committee

J. Villar (Chair), D.G. Altman, L. Cheikh Ismail, R. Craik, S.H. Kennedy, A. Lambert, A.T. Papageorghiou, R. Uauy.

Study Coordinating Unit

J. Villar (Head), S. Ash, R. Craik, L. Cheikh Ismail, S.H. Kennedy, A. Lambert, A.T. Papageorghiou, M. Shorten.

Data Analysis Group

D.G. Altman (Head), E.O. Ohuma, A.T. Papageorghiou, E. Staines Urias, R. Gunier, S. Rauch, J. Villar.

Data Management Group

D.G. Altman (Head), I. Ahmed, S. Ash, C. Condon, M. Mainwaring, D. Muninzwa, M.F. da Silveira, E. Staines Urias, L. Walusuna, S. Wiladphaingern.

Ultrasound Group

A.T. Papageorghiou (Head), L. Salomon (Senior external advisor), M. Buckle, N. Jackson, A. Mitidieri, S. Munim, H. Mwangudzah, R. Napolitano, T. Norris, J. Sande, J. Shah, G. Zainab.

Anthropometry Group

L. Cheikh Ismail (Head), W.C. Chumlea (Senior external advisor), J. Kizidio, B. Monyepote, F. Puglia, M. Salim, R. Salam, V.I. Carrara.

Laboratory Group

R. Craik (Head), D. Alam, Y. Guman, J. Kilonzo, A. Min, V. Ngami, I. Olivera, G. Deutsch.

Neonatal Group

Z.A. Bhutta (Head), E. Bertino, F. Giuliani, R. Uauy.

Environmental Health Group

B. Eskenazi (Head), R. Gunier, S. Rauch.

Neurodevelopment Group

A. Stein (Head), M. Fernandes (Coordinator), A. Abubakar, J. Acedo, L. Aranzeta, L. Cheikh Ismail, F. Giuliani, D. Ibanez, S.H. Kennedy, M. Kihara, E. de Leon, C.R. Newton, S. Savini, A. Soria-Frisch, J. Villar, K. Wulff.

Sapient Metabolomics Group

C. Cheng, K. Dao, L. Glenwinkel, J.M. Gauglitz, T. Long, R. Moranchel, H. Steger, S. Tiwari, J. Watrous, M. Jain.

INTERBIO-21st participating countries and local investigators

Brazil: F.C. Barros (Principal Investigator), M. Domingues, S. Fonseca, A. Leston, A. Mitidieri, D. Mota, I.K. Sclowitz, M.F. da Silveira.

Kenya (Kilifi): J.A. Berkley (Principal Investigator), B. Kemp, H. Barsosio, S. Mwakio, H. Mwangudzah, V. Ngami, M. Salim, A. Seale, L. Walusuna.

Kenya (Nairobi): M. Carvalho and W. Stones (Co-Principal Investigators), D. Muninzwa, J. Kilonzo, J. Kizidio, R. Ochieng, J. Sande, J. Shah.

Pakistan: Z. Bhutta and S. Munim (Co-Principal Investigators), I. Ahmed, D. Alam, A. Raza, R. Salam, G. Zainab.

South Africa: S Norris (Principal Investigator), Y. Guman, T. Lephoto, S. Macauley, L. Malgas.

Thailand: F. Nosten (Principal Investigator), N. Jackson, R. McGready, A. Min, V.I. Cararra, S Wiladphaingern.

UK: S.H. Kennedy (Principal Investigator), S. Ash, M. Baricco, A. Capp, L. Cheikh Ismail, R. Craik, S. Hussein, A. Laister, A. Lambert, T. Lewis, E. Maggiora, R.

Napolitano, T. Norris, A.T. Papageorghiou, B. Patel, F. Puglia, F. Roseman, S. Roseman, M. Sharps, A. Varalda, R. Carew.

Full acknowledgement for all those who contributed to the development of the Project protocol appears at www.interbio21.org.uk
